# Aging triggers an upregulation of a multitude of cytokines in the male and especially the female rodent hippocampus but more discrete changes in other brain regions

**DOI:** 10.1186/s12974-021-02252-6

**Published:** 2021-09-22

**Authors:** Latarsha Porcher, Sophie Bruckmeier, Steven D. Burbano, Julie E. Finnell, Nicole Gorny, Jennifer Klett, Susan K. Wood, Michy P. Kelly

**Affiliations:** 1 Pharmacology, Physiology & Neuroscience, University of South Carolina School of Medicine, 6439 Garners Ferry Rd, Columbia, SC 29209 USA; 2 Department of Anatomy & Neurobiology, University of Maryland School of Medicine, 20 Penn St, HSFII Rm 216, Baltimore, MD 21201 USA; 3 Center for Research on Aging, University of Maryland School of Medicine, 20 Penn St, HSFII Rm 216, Baltimore, MD 21201 USA

**Keywords:** Aged, Aging, Alzheimer’s disease, Cytokine, Chemokine, Inflammatory, Inflammation, Hippocampus, Prefrontal cortex, Sex differences

## Abstract

**Background:**

Despite widespread acceptance that neuroinflammation contributes to age-related cognitive decline, studies comparing protein expression of cytokines in the young versus old brains are surprisingly limited in terms of the number of cytokines and brain regions studied. Complicating matters, discrepancies abound—particularly for interleukin 6 (IL-6)—possibly due to differences in sex, species/strain, and/or the brain regions studied.

**Methods:**

As such, we clarified how cytokine expression changes with age by using a Bioplex and Western blot to measure multiple cytokines across several brain regions of both sexes, using 2 mouse strains bred in-house as well as rats obtained from NIA. Parametric and nonparametric statistical tests were used as appropriate.

**Results:**

In the ventral hippocampus of C57BL/6J mice, we found age-related increases in IL-1α, IL-1β, IL-2, IL-3, IL-4, IL-6, IL-9, IL-10, IL-12p40, IL-12p70, IL-13, IL-17, eotaxin, G-CSF, interfeuron δ, KC, MIP-1a, MIP-1b, rantes, and TNFα that are generally more pronounced in females, but no age-related change in IL-5, MCP-1, or GM-CSF. We also find aging is uniquely associated with the emergence of a module (a.k.a. network) of 11 strongly intercorrelated cytokines, as well as an age-related shift from glycosylated to unglycosylated isoforms of IL-10 and IL-1β in the ventral hippocampus. Interestingly, age-related increases in extra-hippocampal cytokine expression are more discreet, with the prefrontal cortex, striatum, and cerebellum of male and female C57BL/6J mice demonstrating robust age-related increase in IL-6 expression but not IL-1β. Importantly, we found this widespread age-related increase in IL-6 also occurs in BALB/cJ mice and Brown Norway rats, demonstrating conservation across species and rearing environments.

**Conclusions:**

Thus, age-related increases in cytokines are more pronounced in the hippocampus compared to other brain regions and can be more pronounced in females versus males depending on the brain region, genetic background, and cytokine examined.

**Supplementary Information:**

The online version contains supplementary material available at 10.1186/s12974-021-02252-6.

## Background

As humans age, cognitive decline can negatively affect everyday life even in relatively healthy individuals [1, 2]. This cognitive decline is associated with atrophy and reduced plasticity in brain regions such as the hippocampus, cortex, striatum, and, eventually, the cerebellum (c.f [3–5].). In the hippocampus, for example, aging is associated with decreased neurogenesis (e.g., [6]), as well as decreased neuronal size, complexity, and connectivity [1]. The molecular mechanisms underlying this age-related deterioration are not yet fully understood, but increased neuroinflammation is thought to play a role.

Many types of neuroinflammatory and neuroimmune pathways have been implicated in brain aging, including signals related to the Complement Cascade, Toll-like receptor signaling, antigen presentation, and IFϏb/NFϏb signaling as well as macrophage and microglia activation (e.g., [7, 8]). Microglia are the brain’s innate immune cell. Microglia normally reside in a quiescent state until a foreign antigen activates them, at which time they produce pro-inflammatory cytokines (including interleukins, tumor necrosis factors, interferons, and chemokines). Once the threat is resolved, microglia then produce anti-inflammatory cytokines that initiate the return to homeostasis [9]. Within the aging brain, an increase in macrophage infiltration, microglia priming, and/or microglia activation has been observed [9–13], along with a failure to return to homeostasis [9, 11, 14, 15]. Although the early stages of an immune response can be neuroprotective, chronic activation can be detrimental [2, 13, 16, 17]. The chronic activation of microglia that is observed in the aging brain, particularly that within the hippocampus, is thought to cause persistent neuroinflammation that detrimentally affects cognitive function [6, 13, 15, 16, 18–20].

Despite widespread acceptance that persistent neuroinflammation contributes to age-related cognitive decline [13], studies comparing protein expression of cytokines in young versus old brains are surprisingly limited in terms of the number of cytokines and brain regions studied and the fact that most studies only examined effects in either males or females. Studies suggest that aging is associated with increased tumor necrosis factor (TNF) protein expression in the hippocampus and prefrontal cortex [21, 22] but not the amygdala [22]. Although many studies have reported age-related increases in basal interleukin 1β (IL-1β) protein levels in the hippocampus of multiple mouse strains and Wistar rats [23–29], others find no such age-related increases in basal IL-1β protein expression in the hippocampus of F344xBN F1 rats specifically [22, 30–32]. That said, these same reports do find F344xBN F1 rats exhibit age-related exacerbation of IL-1β induction following a challenge [22, 30–32]. Outside of the hippocampus, reports are more limited with studies in SAMR1 mice reporting age-related increases in baseline IL-1β protein expression in the cortex, hypothalamus, and brain stem [29], but studies in C57BL/6J mice and F344xBN F1 rats reporting no such age-related increases in baseline IL-1β protein expression in the cortex, hypothalamus, or amygdala [22, 23, 30, 31]. Findings around IL-6 are even more discrepant. Although 2 studies reported age-related increases in IL-6 protein expression in the hippocampus (BALB/cJ mice, [33]; strain not specified, [21]), 2 other studies did not (C57BL/6J males, [23]; F344xBN F1 Rats, [22]). Similarly, 3 studies reported age-related increases in IL-6 protein expression in the cortex (BALB/cJ mice, [33]; C57BL/6J females, [34]; strain not specified, [21]); however, a fourth did not (C57BL/6J males, [23]).

Discrepancies in the above finding may be related to environmental factors that differ between labs, the brain region examined, the species/strain used in the study, and/or the sex of the subjects employed. For example, diet, immune status (e.g., rearing in a pathogen-free facility), and other environmental factors have been shown to preferentially upregulate cytokine expression in aged vs. young adult brains [11, 12, 35–37]. Pathway analyses suggest that age-related changes in inflammation- and immune-related gene expression may be more pronounced in the hippocampus compared to other brain regions and more pronounced in females versus males [7, 8]. Indeed, we noted that studies employing female subjects alone or in combination with male subjects routinely report higher basal IL-6 mRNA or protein expression in old vs. young brains [8, 34, 38, 39]; whereas, only a subset of studies employing male subjects alone report this age-related increase in basal expression (increased mRNA: [36]; increased protein: [21, 33, 40–42]; no change mRNA: [11, 12, 35, 43]; no change protein: [22, 23, 37]). As such, we sought to clarify how pro-inflammatory (IL-1α, IL-1β, IL-2, IL-3, IL-5 IL-6—see discussion, IL-9, IL-12p40, IL-12p70, IL-17, eotaxin, interfeuron δ, KC, MIP-1a, MIP-1b, rantes, TNFα, MCP-1, or GM-CSF) and anti-inflammatory cytokine expression (IL-4, IL-10, IL-13, G-CSF) may change with age by studying multiple cytokines across multiple brain regions of both male and female subjects, using 2 strains of mice bred in-house as well as rats obtained from the NIA aging colony. Here, we find that in the ventral hippocampus, both pro- and anti-inflammatory cytokines increased with the age in males and even more so in females. Interestingly, while IL-6 was dramatically upregulated with age in all brain regions and subjects examined, IL1-β was only elevated in the ventral and dorsal hippocampus.

## Methods

### Subjects

C57BL/6J and BALB/cJ mice were originally obtained from Jax and then bred onsite at the University of South Carolina School of Medicine. Young mice ranged from 2 to 5 months old, middle-aged mice ranged from 9 to 12 months old, and old mice ranged from 18 to 24 months old. Litter effects are unlikely to account for effects described herein as mice were derived from multiple litters and were housed across multiple cages. Further, several sample sets analyzed by Western blot were obtained from multiple cohorts of mice born and raised at different times in our facility, with some cohorts including young, middle-aged and old mice, and other cohorts including only young and old mice. Young (4 months old) and old (19 months old) Brown Norway Rats were obtained directly from the NIA colony and allowed to acclimate to the animal facility at the University of South Carolina School of Medicine for 1 week prior to tissue harvest. Animals are housed on a 12:12 light:dark cycle and allowed ad lib access to food and water. Experiments were carried out in accordance with the National Institutes of Health Guide for the Care and Use of Laboratory Animals (Pub 85-23, revised 1996) and were fully approved by the Institutional Animal Care and Use Committee of the University of South Carolina and the University of Maryland, Baltimore. See Table 1 for specific n’s in each experiment. Note that males and females were used throughout all experiments but not necessarily in sufficient number to power an analysis of age x sex. Mice are generally healthy at the time of tissue harvest. We do not conduct gross pathology; however, mice are routinely assessed by husbandry, veterinary, and laboratory staff and mice with palpable tumors, lethargy, altered gait, signs of malnutrition, or dehydration are removed from the study and euthanized. If mice were to demonstrate evidence of a striking anatomical abnormality of the brain upon dissection (e.g., a pituitary tumor), they would also not be included since it is our intent is to study the effects of healthy aging. Note that a previous study found ~35% of C57BL6 female mice raised in a pathogen-free facility exhibited pituitary tumors [44]; however, we came upon only a handful of these readily identifiable tumors (or tumors of any sort) in the brains of our C57BL/6J mice raised in a conventional animal facility.

**Table 1:** Statistics and *n*’s for experiments shown in Figs. 1, 2, 3, 4, and 5

	YM	YF	MM	MF	OM	OF				
Figure			***n***=				Normality	EV^**a**^	effect of age	Effect of sex^b^
1A	4	3			4	3	Pass	Fail	Rank sum: *T*(7,7)=35.00, FDR-P=0.036	
1B	4	3			4	3	Pass	Fail	Rank sum: *T*(7,7)=30.50, FDR-P=0.013	
1C	4	3			4	3	Pass	Pass	Student’s *t*: *t*(12)=-3.472, FDR-P=0.019	
1D	4	3			4	3	Pass	Pass	Student’s *t*: *t*(12)=-2.375, FDR-P=0.044	
1E	4	3			4	3	Pass	Pass	Student’s *t*: *t*(12)=-3.128, FDR-P=0.023	
1F	2	1			2	1	Pass	Pass	Student’s *t*: *t*(4)=-1.53, FDR-P=0.178	
1G	4	3			4	3	Pass	Fail	Rank sum: *T*(7,7)=31.00, FDR-P=0.019	
1H	4	3			4	3	Pass	Pass	Student’s *t*: *t*(12)=-2.340, FDR-P=0.044	
1I	4	3			4	3	Fail		Rank sum: *T*(7,7)=32.00, FDR-P= 0.023	
1J	4	3			4	3	Fail		Rank sum: *T*(7,7)=32.00, FDR-P=0.023	
1K	4	3			4	3	Pass	Pass	Student’s *t*: *t*(12)=-5.641, FDR-P=0.002	
1L	4	3			4	3	Fail		Rank sum: *T*(7,7)=28.50, FDR-P=0.007	
1M	4	3			4	3	Pass	Fail	Rank sum: *T*(7,7)=34.00, FDR-P=0.029	
1N	4	3			4	3	Fail		Rank sum: *T*(7,7)=28.00, FDR-P=0.007	
1O	4	3			4	3	Fail		Rank sum: *T*(7,7)=33.50, FDR-P=0.025	
1P	4	3			4	3	Pass	Pass	Student’s *t*: *t*(12)=−1.052, FDR-P=0.314	
1Q	4	3			4	3	Pass	Pass	Student’s-*t: t*(12)=-2.386, FDR-P=0.044	
1R	4	3			4	3	Pass	Pass	Student’s *t*: *t*(12)=-2.996, FDR-P=0.025	
1S	4	3			4	3	Fail		Rank sum: *T*(7,7)=41.00, FDR-P=0.179	
1T	4	3			4	3	Pass	Pass	Student’s *t*: *t*(12)=-2.554, FDR-P=0.036	
1U	4	3			4	3	Fail		Rank sum: *T*(7,7)=33.00, FDR-P=0.025	
1V	4	3			4	3	Pass	Pass	Student’s *t*: *t*(12)=-2.836, FDR-P=0.027	
1W	4	3			4	3	Pass	Fail	Rank sum: *T*(7,7)=34.50, FDR-P=0.026	
2A-left	7	7	4	4	8	6	Fail		ANOVA on ranks: *H*(2)=1.51, *P*=0.471	Rank sum: *T*(6,8)=47.0, *P*=0.852
2A-right	7	7	4	4	8	6	Pass	Fail	ANOVA on ranks: *H*(2)=8.505, *P*=0.014^c^	Rank sum: *T*(5,8)=41.0, *P*=0.435
2B	13	13	17	16	16	13	Fail		ANOVA on ranks: *H*(2)=44.227, *P*<0.001^c^	Rank sum: *T*(13,16)=252.00, *P*=0.013
2C-left	9	5			9	4	Pass	Fail	Rank sum: *T*(13,14)=226.00, *P*=0.035	Rank sum: *T*(4,9)=31.0, *P*=0.70
2C-right	9	5			9	4	Pass	Fail	Rank sum: *T*(13,14)=247.00, P=0.002	Rank sum: *T*(4,9)=30.0, *P*=0.817
3A	9	8	12	11	17	14	Fail		ANOVA on Ranks: H(2) = 50.059, *P*<0.001^c^	Rank sum: *T*(14,17)=230.0, *P*=0.827
3B-left	6	5			5	5	Pass	Pass	2-Way ANOVA: *F*(1,17)=11.207, *P*=0.004	2-Way ANOVA: *F*(1,17)=0.01, *P*=0.928
3B-right	6	5			5	5	Pass	Pass	2-Way ANOVA: *F*(1,17)=11.443, *P*=0.004	2-Way ANOVA: *F*(1,17)=0.003, *P*=0.955
3C	15	12			9	14	Fail		Rank sum: *T*(23,27)=289.0, *P*<0.001	Rank sum: *T*(4,8)=23.0, *P*=0.683
3D-left	2	3			2	3	Pass	Pass	Student’s *t*: *t*(8)=0.43, *P*=0.677	
3D-right	2	3			2	3	Pass	Pass	Student’s *t*: *t*(8)=0.18, *P*=0.862	
3E	16	13			10	10	Pass	Pass	2-Way ANOVA: *F*(1,45)=74.93, P<0.001	2-Way ANOVA: *F*(1,16)=0.22, *P*=0.649
3F-left	11	6		11	7	Fail			Rank sum: *T*(17,18)=270.5, P=0.248	Rank sum: *T*(7,11)=50.0, *P*=0.147
3F-right	11	7		11	7	Pass	Fail		Rank sum: *T*(18,18)=328.0, P=0.887	Rank sum: *T*(7,11)=46.0, *P*=0.07
3G	14	13	16	17	16	16	Fail		ANOVA on ranks: *H*(2)=53.334, *P*<0.001^c^	Rank sum: *T*(15,16)=207.0, *P*=0.199
3H	9	9	4	4	9	9	Pass	Pass	2-Way ANOVA: *F*(2,38)=1.90, *P*=0.163	2-Way ANOVA: *F*(1,6)=0.37, *P*=0.564
4A	2	5			2	5	Pass	Fail	Rank sum: *T*(7,7)=30.0, *P*=0.002	
4B	2	5			2	5	Fail		Rank sum: *T*(7,7)=28.0, *P*<0.001	
4C	2	5			2	5	Fail		Rank sum: *T*(7,7)=29.0, *P*=0.001	
4D	2	5			2	5	Fail		Rank sum: *T*(7,7)=28.0, *P*<0.001	
4E	2	5			2	5	Pass	Pass	Student’s t: *t*-test: *t*(12)=-4.01, *P*=0.002	
4F	5	5			5	5	Pass	Pass	2-Way ANOVA: *F*(1,16)=55.42, *P*<0.001	2-Way ANOVA: *F*(1,16)=0.002, *P*=0.966
4G	5	5			5	5	Pass	Pass	2-Way ANOVA: *F*(1,16)=55.42, *P*<0.001	2-Way ANOVA: *F*(1,16)=14.36, *P*=0.002
5A-nuc	7	2			6	2	Pass	Fail	Rank sum: *T*(8,9)=108.0, *P*<0.001	
5A-cyto	7	2			6	2	Fail		Rank sum: *T*(8,9)=108.0, *P*<0.001	
5A-memb	7	2			6	2	Fail		Rank sum: *T*(8,9)=105.0, *P*=0.002	
5B-nuc	7	2			6	2	Pass	fail	Rank sum: *T*(8,9)=107.0, *P*<0.001	
5B-cyto	7	2			6	2	Fail		Rank sum: *T*(8,9)=105.0, *P*=0.002	
5B-memb	6	2			6	2	Fail		Rank sum: *T*(8,8)=36.0, *P*<0.001	

*YM* young males, *YF* young females, *MM* middle-aged males, *MF* middle-aged females, *OM* old males, *OF* old females, *EV* equal variance, *FDR* corrected for multiple comparisons using false detection rate

^a^Equal variance only tested when dataset passed normality

^b^Effect of sex only analyzed in experiments where *n*>4/sex/age. Rank sum used when 2-way ANOVA for age x sex failed normality or equal variance and reports effect of sex within old mice. 2-way ANOVA used when normality and equal variance pass and reports main effect of sex across ages

^c^See the “Results” section for post hoc tests

### Tissue collection

Mice were euthanized during the light cycle via cervical dislocation, whilst rats were euthanized during the light cycle by CO_2_ inhalation. Mouse brains were harvested fresh, dissected on wet ice, and stored at −80°C for further processing. Rat brains were harvested fresh, hemisected over wet ice, frozen in isopentane over dry ice, and half brains were then stored at −80°C. Later, the cerebellum and prefrontal cortex were dissected from frozen rat half brains on dry ice. All brain regions examined in the BALB/cJ mice were dissected from the same group of animals.

### Bioplex

A Bio-Plex Pro Mouse Cytokine 23-Plex (Bio-Rad, Hercules CA) was used to probe ventral hippocampal homogenates for cytokines. Methods for the tissue preparation were based on previously published work in rat brain homogenates [45, 46]. Briefly, ventral hippocampal samples were homogenized in boiling lysis buffer (50-mM NaF/1% SDS [47];), and then, homogenates were diluted in sample diluent containing 1% FBS. Standards were reconstituted in lysis buffer containing 1% FBS and diluted via serial dilution such that all analytes reached a minimum concentration of 0.2 pg/mL. Bioplex data were collected over 3 runs using the same plate. In the first run, ½ of the wells were used to test all samples at a concentration of 0.275 μg/μL based on our previous work with rat tissue [45, 46]. Unfortunately, many targets were unable to be detected due to matrix effects or reached ceiling effects at that high of a concentration (See Table S1). In the 2nd run, we used approximately ¼ of the wells to retest ½ of these samples at a concentration of 0.1 μg/μL. We found 0.1 μg/μL allowed detection of all targets except IL-5. Therefore, in a 3rd run we used the remaining ¼ of the plate to retest the other ½ of the samples at a concentration of 0.05 μg/μL. 0.05 μg/μL enabled detection of all targets. The pattern of effects was consistent across the subsets of samples tested at 0.1 μg/μL and 0.05 μg/μL. As such, data from each concentration were expressed as a fold change of the young mean and combined in analyses. Bead preparation, handling, and plate processing were conducted according to manufacturer protocol. Plates were washed using a Bio-Plex Pro II Wash Station (Bio-Rad, Hercules, CA) and read using a Luminex SD system (Bio-Rad, Hercules CA) housed within the Instrument Resource Facility at the University of South Carolina School of Medicine.

### Western blots

Samples for select C57BL/6J Westerns, BALB/cJ Westerns, and rat Westerns were homogenized using a sonic dismembrator (a.k.a. tissue sonicator), as previously described [47, 48], in boiling lysis buffer (50 mM NaF/1% SDS). The tissue for the remaining C57BL/6J Westerns was homogenized as previously described [47] in ice-cold lysis buffer (20 mM Tris-HCl, pH 7.5; 2 mM MgCl_2;_ Thermo Pierce Scientific phosphatase tablet #A32959 and protease inhibitor 3 #P0044) in preparation for subsequent biochemical fractionation of the samples. There were no differences in the pattern of Western results obtained with one or the other lysis buffer, and so data were combined. Total protein quantity was determined for all homogenized tissue using the DC Protein Assay kit (BioRad, Inc.; Hercules, CA), and western blots were carried out as previously outlined [47, 48]. For all blots, 36.3 μg of total protein or 22 μg of fractionated protein was loaded onto 4–12% Bis-Tris gels (Life Technologies) for electrophoresis. Following transfer to nitrocellulose membranes (#10600008, Amersham), all blots except those testing the biochemical fractionations were subjected to PonceauS (#6266-79-5, Fisher Scientific) to stain the total protein in each sample. Following image capture, The PonceauS was washed off with ultrapure water and TBST and the membranes were cut according to the molecular weight of the target of interest (Figure S1). Superblock (#37515, Thermofisher) was then used at room temperature to block non-specific binding sites on membranes, and membranes were subsequently probed overnight at 4°C with primary antibodies against IL-6 (early experiments with 1:200 of MAB406 from R&D Systems; later experiments with 1:2500 of ARX0962 from Life Technologies), IL-1β (ab106034 from Abcam, 1:5,000), IL-10 (ARG2419 from Arigo Biolaboratories, 1:500), and actin (A2066 from Sigma, 1:10,000) in Pierce Superblock (P137517)/0.1% Tween20 (Fisher BP337-500). The following day, blots were washed in TBST and incubated for 1 h at room temperature with a species-specific HRP-tagged secondary antibody (Jackson, 1:10,000). Blots probed with IL-6 and IL-1β were developed using WesternSure Premium Chemiluminescent Substrate (926-95000), whereas blots probed with actin were developed using Pierce SuperSignal West Pico CL Substrate (#34078). Blots were then apposed to film, scanned in at 1200 dpi, and quantified by densitometry using ImageJ (NIH) by a group-blinded experimenter. Data from the biochemical fractions were normalized to actin as a loading control as these data were obtained several years prior to the lab implementing the use of PonceauS. All other Western data were normalized to PonceauS staining intensity as a loading control. Unadjusted images of the full blots and membranes from which cropped images were pulled are shown in Figures S1-S8.

### Data analysis

Biochemical data were collected by an experimenter blind to treatment, and Sigmaplot 11.2 was used to analyze data. As described above, Bioplex data were obtained from 2 separate runs, each using a different concentration of tissue. Therefore, Bioplex data from each run were normalized to the young group from that run. Similarly, Western data for a given experiment span multiple gels and so are normalized to the Young group on each gel in order to mitigate any non-specific differences between blots related to transfer efficiencies, film exposures, etc. (as in [47, 48]). For Westerns, each brain region and fraction were run on separate sets of blots and so resultant data are analyzed with individual statistical tests per region or fraction. Both males and females were included in each group, but not always in sufficient number to analyze for an effect sex. For experiments with fewer than *n*=4/sex/group, data were analyzed for age only. For experiments that included at least *n*=4/sex/group, data were analyzed for both age and sex. Parametric statistics were used (i.e., 2-factor ANOVA or Student *t* test) when datasets passed normality (Shapiro-Wilk test) and equal variance (Levene’s test). In cases where 2-factor ANOVAs failed assumptions of normality and/or equal variance, statistical tests for each factor were conducted separately. When statistical tests failed normality and/or equal variance, a nonparametric Whitney rank sum test or Kruskal-Wallis ANOVA on ranks was used. Correlations were conducted using Spearman rank order. Given all Bioplex data were derived from the simultaneous measurement of multiple endpoints, a false-rate discovery (FDR) correction was applied to all *P* values to mitigate the risk of type I error associated with multiple comparisons. Outliers >2 standard deviations from the mean were removed from analyses consistent with our previous publications (e.g., [47, 49]; outliers/total data points: Fig. 2A, 3/72; Fig. 3F, 1/72; Fig. 3G, 3/92).

**Fig. 1:**
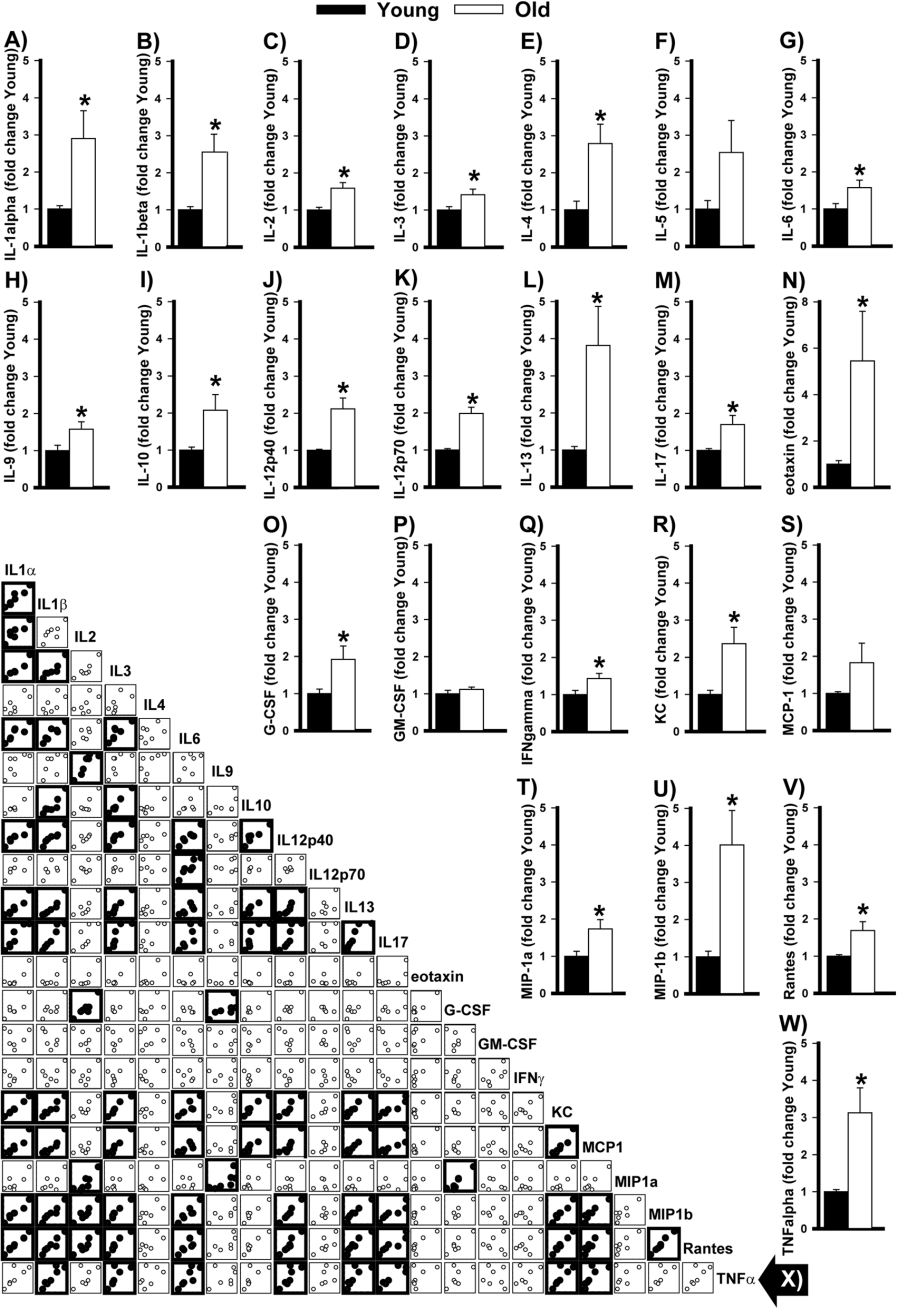
Bioplex analyses show widespread upregulation of cytokines in old versus young C57BL/6J ventral hippocampus. Cytokine expression was measured in the ventral hippocampus of young versus old C57BL/6J. Relative to young mice, old mice expressed significantly higher levels of **A** interleukin 1α (IL-1α), **B** IL-1β, **C** IL-2, **D** IL-3, and **E** IL-4 but not **F** IL-5. Old mice also expressed higher levels of **G** IL-6, **H** IL-9, **I** IL-10, **J** IL-12p40, **K** IL-12p70, **L** IL-13, **M** IL-17, **N** eotaxin, and **O** granulocyte colony-stimulating factor (G-CSF), but not **P** granulocyte-macrophage colony-stimulating factor (GM-CSF). **Q** Interfeuron gamma (IFNγ) also exhibited an age-related increase, as did **R** KC but not **S** monocyte chemoattractant protein 1 (MCP1). Finally, **T** macrophage inflammatory protein 1a (MIP-1a), **U** MIP-1b, **V** rantes, and **W** tumor necrosis factor (TNFα) all demonstrated age-related increases in expression. **X** Correlational analyses (bold = FDR-P<0.05) of data from old mice reveals that a subset of these cytokines become uniquely coupled with each other in the aged ventral hippocampus (i.e., IL-1α, IL-1β, IL-3, IL-6, KL12p40, IL-13, IL17, KC, MCP-1, MIP-1b and Rantes; see Table S2 for *r* and *P* values for both young and old mice). Data expressed as mean ±SEM. *vs. young, FDR-P=0.044–0.002. FDR—false discovery rate

## Results

### Bioplex reveals pervasive upregulation of cytokines with age in the ventral hippocampus

To determine if the ventral hippocampus exhibits a widespread upregulation of cytokines with age, we used a Bioplex to simultaneously measure 23 such endpoints in young versus old C57BL/6J mice. Relative to young mice, old mice expressed significantly higher levels of IL-1α, IL-1β, IL-2, IL-3, IL-4, IL-6, IL-9, IL-10, IL-12p40, IL-12p70, IL-13, IL-17, eotaxin, granulocyte colony-stimulating factor, interferon δ, KC, macrophage inflammatory protein 1a (MIP-1a), MIP-1b, rantes, and TNFα (Fig. 1; Table 1). The age-related increases in the above noted pro-inflammatory and anti-inflammatory cytokines occurred in equal proportion, with only the ratio of IL-1β/IL-13 differing significantly between old and young mice (Table 2). Effects noted in females tended to be stronger than those noted in males (Figure S9); however, the study was not powered for an analysis of sex effects (see Western data below). The age-related increases in IL-5 and monocyte chemoattractant protein 1 failed to reach the level of statistical significance and granulocyte-macrophage colony-stimulating factor showed no difference in expression between young and old mice. In addition to expression changes, aging was also associated with the emergence of novel correlations in expression that were not observed in young mice. Whereas young mice only showed 9 significant correlations (no more than 3 for a given cytokine), old mice showed a total of 80 significant correlations (Table S2; Figure 1X). Interestingly, these significant correlations in old mice were not randomly distributed. Rather, 65/80 significant correlations fell within a module (i.e., a cluster of cytokines whose signals strongly correlate with each other [50]) of 11 cytokines (IL-1α, IL-1β, IL-3, IL-6, KL12p40, IL-13, IL17, KC, MCP-1, MIP-1b, and Rantes; Table S3). The degree of correlation was not related to the effect size of the age-related increases, with cytokines showing no, small or large changes represented amongst both the highly and rarely correlated cytokines. The degree of correlation was also not related to the designation of anti- vs pro-inflammatory, with members of each family amongst the highly versus rarely correlated cytokines. Among the highly correlated cytokines, IL-1β, IL-3, IL-6, KL12p40, IL-13, IL17, KC, and MCP-1 formed a particularly tightly coupled core module, with each correlated to the other. Taken together, these data suggest the ventral hippocampus exhibits a dramatic change in the regulation of cytokines with age.

**Table 2:** Ratios of pro/anti-inflammatory cytokines suggest both types of cytokines are largely upregulated in the ventral hippocampus of old versus young C57BL/6J mice to the same extent

Ratio^**a**^	Young mean	Young SEM	Old mean	Old SEM	Student’s ***T*** test or rank sum	Raw ***P*** value	FDR-P value
IL-1α/IL-10	1.00	0.05	1.30	0.18	Failed EV: *T*(7,7)=39.00	0.0973	0.37537
IL-1β/IL-10	1.01	0.06	1.25	0.13	*t*(12)=-1.705318	0.1139	0.38428
IL-2/IL-10	1.04	0.12	0.85	0.08	*t*(12)=1.317512	0.2123	0.4776
IL-6/IL-10	1.03	0.08	1.09	0.12	*t*(12)=-0.369068	0.7185	0.84346
IL-12p40/IL-10	1.04	0.09	1.09	0.09	*t*(12)=-0.391351	0.7024	0.90308
IL-12p70/IL-10	1.05	0.12	1.09	0.14	*t*(12)=-0.232436	0.8201	0.88573
G-CSF/IL-10	1.01	0.10	0.95	0.08	*t*(12)=0.469474	0.6471	0.87365
IFNγ/IL-10	1.03	0.12	0.80	0.14	*t*(12)=1.204295	0.2517	0.48541
MIP-1α/IL-10	1.13	0.29	0.91	0.10	Failed normality: *T*(7,7)=55.00	0.8048	0.90538
TNFα/IL-10	1.02	0.05	1.53	0.28	Failed normality: *T*(7,7)=37.00	0.0530	0.28636
IL-1α/IL-4	1.00	0.18	1.06	0.20	*t*(12)=-0.21	0.8382	0.87044
IL-1β/IL-4	1.00	0.19	0.98	0.15	*t*(12)=0.10	0.9183	0.9183
IL-2/IL-4	0.99	0.20	0.66	0.11	*t*(12)=1.46	0.1727	0.4239
IL-6/IL-4	1.02	0.17	0.87	0.16	*t*(12)=0.64	0.5300	0.795
IL-12p40/IL-4	0.99	0.15	0.86	0.13	*t*(12)=0.64	0.5400	0.76737
IL-12p70/IL-4	0.96	0.15	0.87	0.17	*t*(12)=0.38	0.7100	0.87136
G-CSF/IL-4	1.07	0.22	0.76	0.13	*t*(12)=1.30	0.2200	0.45692
IFNg/IL-4	1.02	0.26	0.60	0.10	*t*(12)=1.59	0.1400	0.42
MIP-1α/IL-4	0.92	0.09	0.68	0.08	*t*(12)=2.06	0.0636	0.2862
TNFα/IL-4	0.99	0.16	1.22	0.26	*t*(12)=-0.72	0.4844	0.76934
IL-1β/IL6	1.00	0.06	1.19	0.10	Failed normality: *T*(7,7)=16.0	0.3200	0.576
GM-CSF/IL-6	1.01	0.10	0.62	0.12	*t*(12)=2.563987	**0.0248**	0.33506
IFNγ/IL-6	1.01	0.11	0.76	0.13	*t*(12)=1.451187	0.1724	0.46538
IL-1α/IL-13	1.01	0.06	0.76	0.09	*t*(12)=2.445990	**0.0308**	0.27736
IL-1β/IL-13	1.02	0.05	0.74	0.05	*t*(12)=3.973891	**0.0018**	**0.04987**
MIP-1α/IL-13	1.15	0.33	0.58	0.11	Failed normality: *T*(7,7)=68.0	0.0530	0.35775
TNFα/IL-13	1.05	0.11	0.89	0.12	*t*(12)=1.02	0.3286	0.55451

*EV* equal variance

^**a**^Pairings of pro- vs anti-inflammatory cytokines based on https://www.sinobiological.com/resource/cytokines/all-anti-inflammatory-cytokines (accessed 04/05/21). Note that 1 young subject expressed no IL-4; therefore, data from this subject could not be expressed as a ratio. Thus, *n*=7/age for all except for IL-4 ratios where *n*=6 for young

### Western blots confirm age-related increases in ventral hippocampal cytokines

**Fig. 2:**
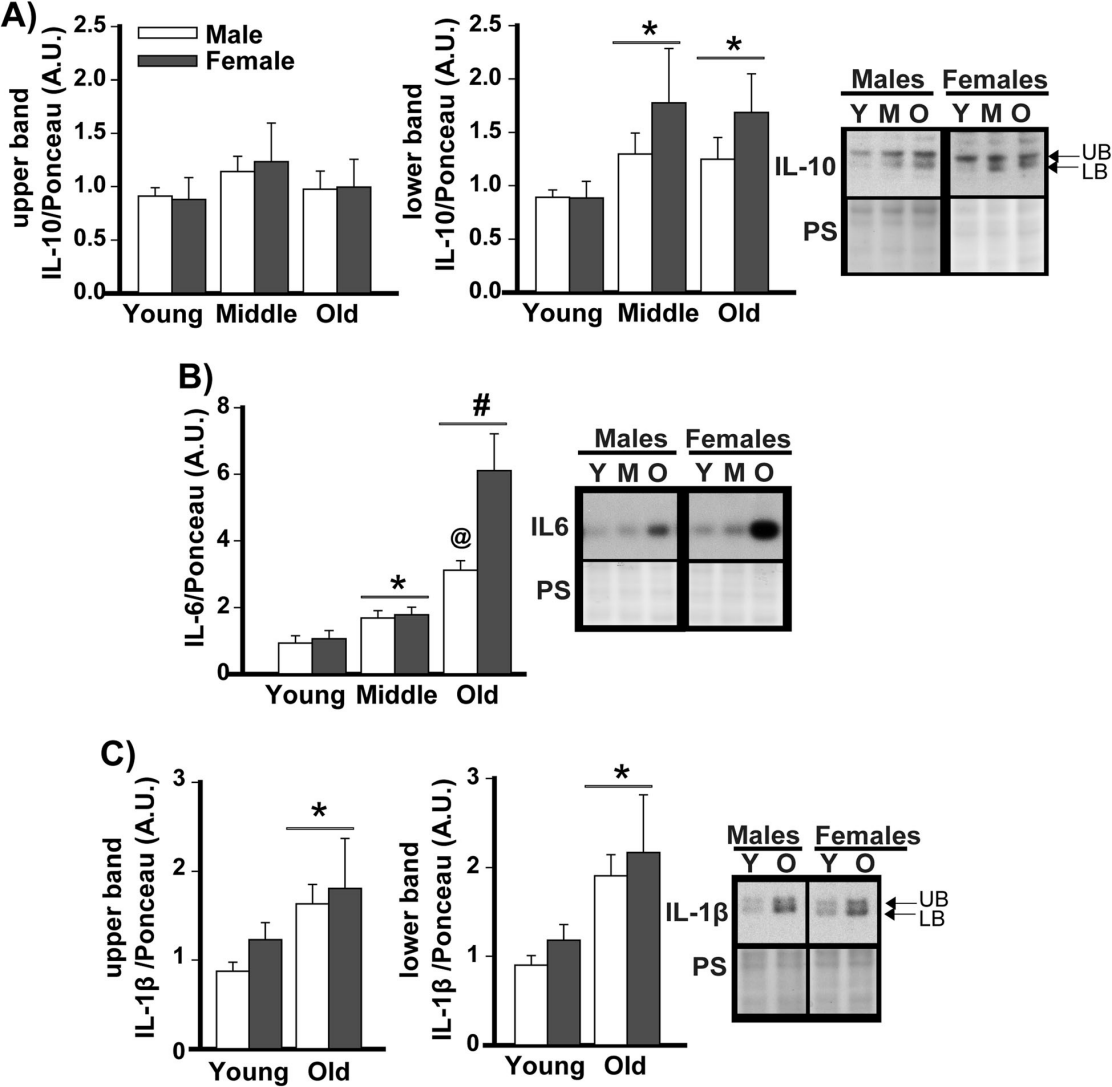
Western blots confirm IL-10, IL-6, and IL-1β expression increase with age in mouse ventral hippocampus. **A** An IL-10 antibody detected a doublet migrating at ~25 kDa, possibly due to detection of differentially glycosylated forms. Across sexes, old and middle-aged mice showed higher expression of the bottom band relative to young adult mice. The fact that the effect of age occurs across sexes is indicated by the significance marker (i.e., * or #) being placed above a bar that extends across the male and female histograms (see Table 1). **B** IL-6 (migrating as a single band at ~21 kDa) was also elevated in old mice relative to young adult and middle-aged mice. This age-related increase was observed across males and females, but was smaller in males. **C** Across sexes, IL-1β protein (migrating as a doublet at ~25 kDa possibly due to detection of differentially glycosylated forms) was also elevated in old versus young adult mice. Data expressed as mean ±SEM. Brightness and contrast of blots and Ponceau stain (PS) images adjusted for graphical clarity. Effect of age across sexes: *vs. young, *P*=0.049 to <0.00001; #vs. young and middle-aged, *P*<0.001. Rank Sum test of sex within old: @vs. female, *P*=0.013. LB—lower band, UB—upper band, Y—young, M—middle aged, O—old.

To confirm results obtained from the Bioplex and further investigate potential sex effects, IL-10, IL-6, and IL-1β expression in the ventral hippocampus were assessed using Western blots. In the ventral hippocampus of C57BL/6J mice, expression of presumed unglycosylated IL-10 (i.e., lower band on Western blot) appears to increase with age across males and females, whereas expression of presumed glycosylated IL-10 (i.e., upper band on Western blot) appears to remain stable (Fig. 2A; see Table 1 for statistics; post hoc: young vs. middle age *P*=0.035, young vs. old *P*=0.046). As such, the ratio of glycosylated/unglycosylated IL-10 decreased with age (Figure S10A). In contrast, both presumed unglycosylated and glycosylated forms of IL-1β increased in expression with age in both males and females (Fig. 2C; see Table 1 for statistics). Even though both isoforms of IL-1β increased with age, the ratio of glycosylated/unglycosylated IL-1 β still decreased with age as was seen with IL-10 (Figure S10B). IL-6 expression also increased across the lifespan of male and female C57BL/6J mice (Fig. 2B; see Table 1 for statistics; post hoc: young vs. middle-aged *P*=0.049, young vs. old *P*<0.001; middle-aged vs old *P*<0.001), but females showed a much larger age-related increase than males. Together, these data support a widespread upregulation of cytokines in the ventral hippocampus with age.

### Western blots show age-related increases in cytokines are more discreet in extrahippocampal brain regions

**Fig. 3:**
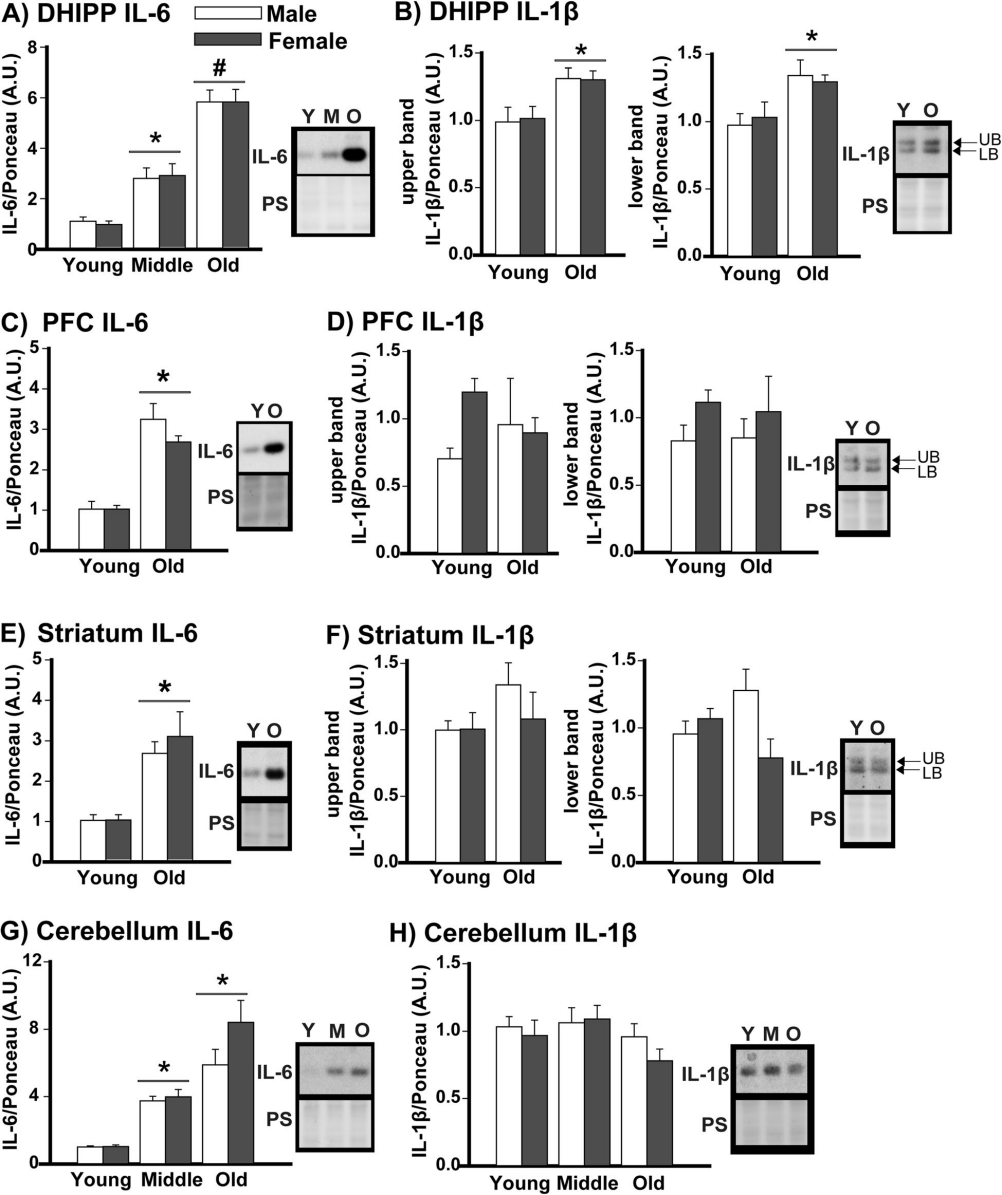
Age-related increases in IL-1β appear to be restricted to the hippocampus, whereas age-related increases in IL-6 are noted throughout the brain. **A** Across sexes, middle-aged, and old mice expressed higher levels of IL-6 protein (migrating at ~21 kDa) relative to young mice in the dorsal hippocampus. The fact that the effect of age occurs across sexes is indicated by the significance marker (i.e., * or #) being placed above a bar that extends across the male and female histograms (see Table 1). **B**) IL-1β (migrating as a doublet at ~25 kDa) also appears to be upregulated with age across sexes in the dorsal hippocampus as old mice showed stronger signals in both the upper and lower bands relative to young mice. **C** IL-6 was significantly elevated in the prefrontal cortex of old mice relative to young mice; however, **D** IL-1β expression did not change with age in the prefrontal cortex. A similar pattern was observed in striatum, with **E** old mice expressing more IL-6 than young mice but **F** not more IL-1β. **G** In the cerebellum, both middle-aged and old mice showed higher levels of IL-6 relative to young mice. **H** In contrast, IL-1β—which migrated as a single-thick band in the cerebellum—did not change with age. Data expressed as mean ±SEM. Brightness and contrast of blots and Ponceau stain (PS) images adjusted for graphical clarity. Effect of age across sexes: *vs young, *P*=0.005 to <0.001; #vs young and middle, *P*<0.001; rank sum test of sex within old: @vs. male, *P*=0.044. LB—lower band, UB—upper band, Y—young, M—middle aged, O—old

To determine the extent to which cytokines are upregulated with age in the brain, Western blots were used to analyze IL-6 and IL-1β in the dorsal hippocampus, prefrontal cortex (PFC), striatum, and cerebellum of C57BL/6J mice. IL-6 expression increased with age in the dorsal hippocampus (Fig. 3A, see Table 1 for statistics; post hoc: young vs middle *P*=0.005, young vs. old *P*<0.001, middle vs. old *P*<0.001), prefrontal cortex (Fig. 3C; Table 1), striatum (Fig. 3E; Table 1), and cerebellum of mice (Fig. 3G; Table 1; post hoc: middle age or old vs. young, *P*<0.001). In contrast, IL-1β expression only increased with age in the dorsal hippocampus (Fig. 3B; Table 1). Unlike the ventral hippocampus, there was no age-related decrease in glycosylated/unglycosylated IL-1β in the dorsal hippocampus (Figure S10C) or prefrontal cortex (Figure S10D), and females actually showed an increase in glycosylated/unglycosylated IL-1β in the striatum (Figure S10E). Also unlike the ventral hippocampus, males and females showed equivalent age-related increases in IL-6 expression in the dorsal hippocampus, prefrontal cortex, striatum, and cerebellum suggesting the heightened age-related increases in IL-6 observed above in females are restricted to the ventral hippocampus in this mouse strain. Age-related increases in IL-6 expression outside of the hippocampus were confirmed in the second cohort of C57BL/6J mice with middle-aged mice (*n*=5M/6F) showing higher expression relative to young mice (*n*=5/sex) in the prefrontal cortex (young, 0.228 ±0.01 A.U.; middle, 1.00 ±0.07 A.U.; rank sum test: *T*(10,11)=55.00, *P*<0.001) and striatum (young, 0.275 ±0.03 A.U.; middle, 1.00 ±0.07 A.U.; rank sum test: *T*(10,11)=45.00, *P*<0.001). These data together with the Bioplex data suggest that while the hippocampus exhibits a widespread upregulation of cytokines with age, other brain regions exhibit a more restricted upregulation.

### Western blots show age-related increases in IL-6 are also observed in BALB/cJ mice and Brown Norway Rats

**Fig. 4:**
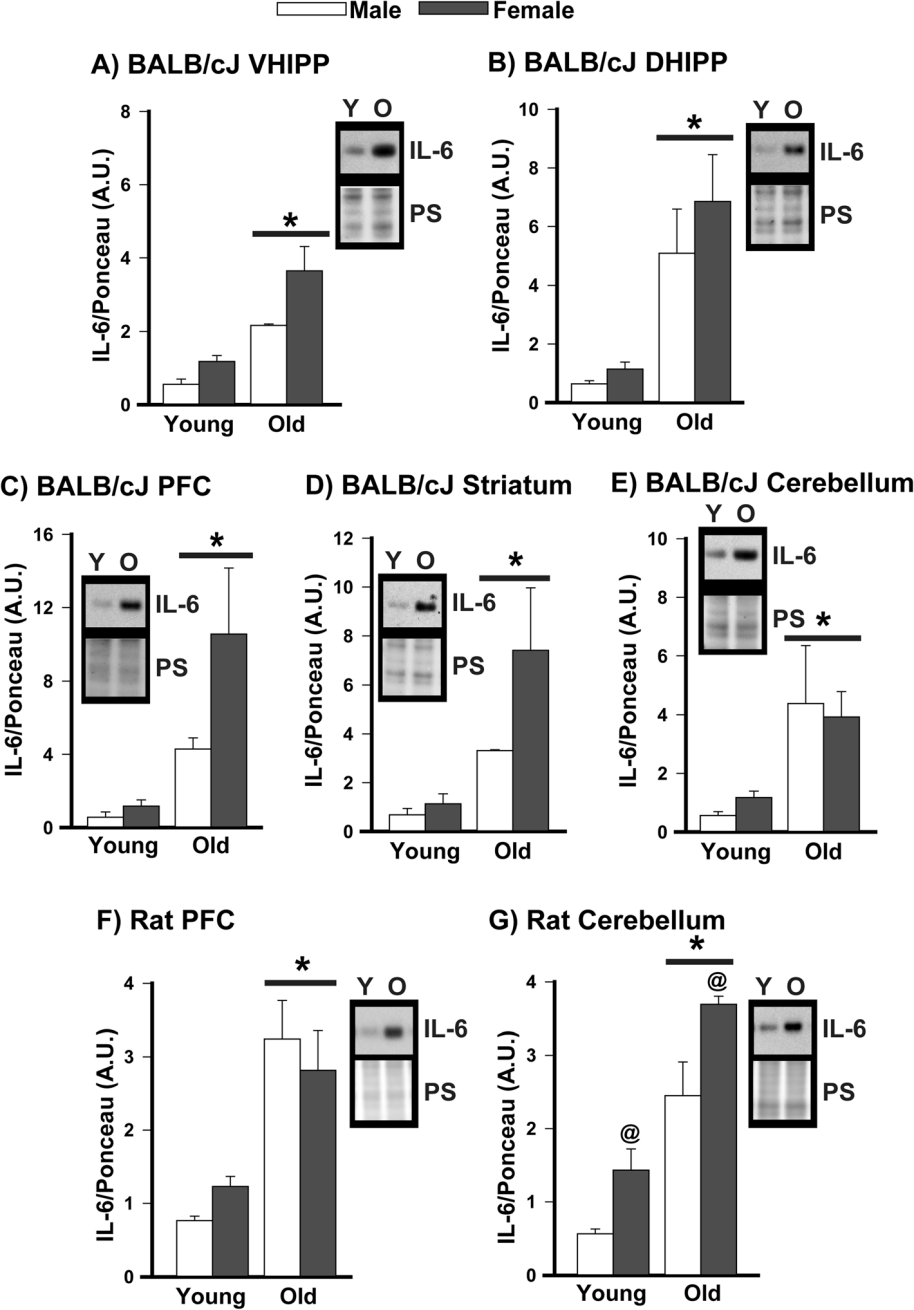
Age-related increases in IL-6 are also noted in multiple brain regions of BALB/cJ mice and Brown Norway Rats. Relative to young BALB/cJ mice, old BALB/cJ mice express significantly higher levels of IL-6 protein in the **A** ventral hippocampus (VHIPP; effect of age across sexes indicated by the bar extended across the male and female histograms), **B** dorsal hippocampus (DHIPP), **C** prefrontal cortex (PFC), **D** striatum, and **E** cerebellum. Similarly, old Brown Norway Rats showed higher expression of IL-6 protein relative to young rats in the **F** PFC and **G** cerebellum, with females expressing higher levels than males in the cerebellum across ages. These data suggest widespread age-related increases in IL-6 expression are conserved across species and are not tied to a single rearing environment. Data expressed as mean ±SEM. Brightness and contrast of blots and Ponceau stain (PS)**.** images adjusted for graphical clarity. *Main effect of age (vs. young), *P*=0.002 to <0.001; @main effect of sex (vs. male), *P*=0.002. Y—young, O—old

**Fig. 5:**
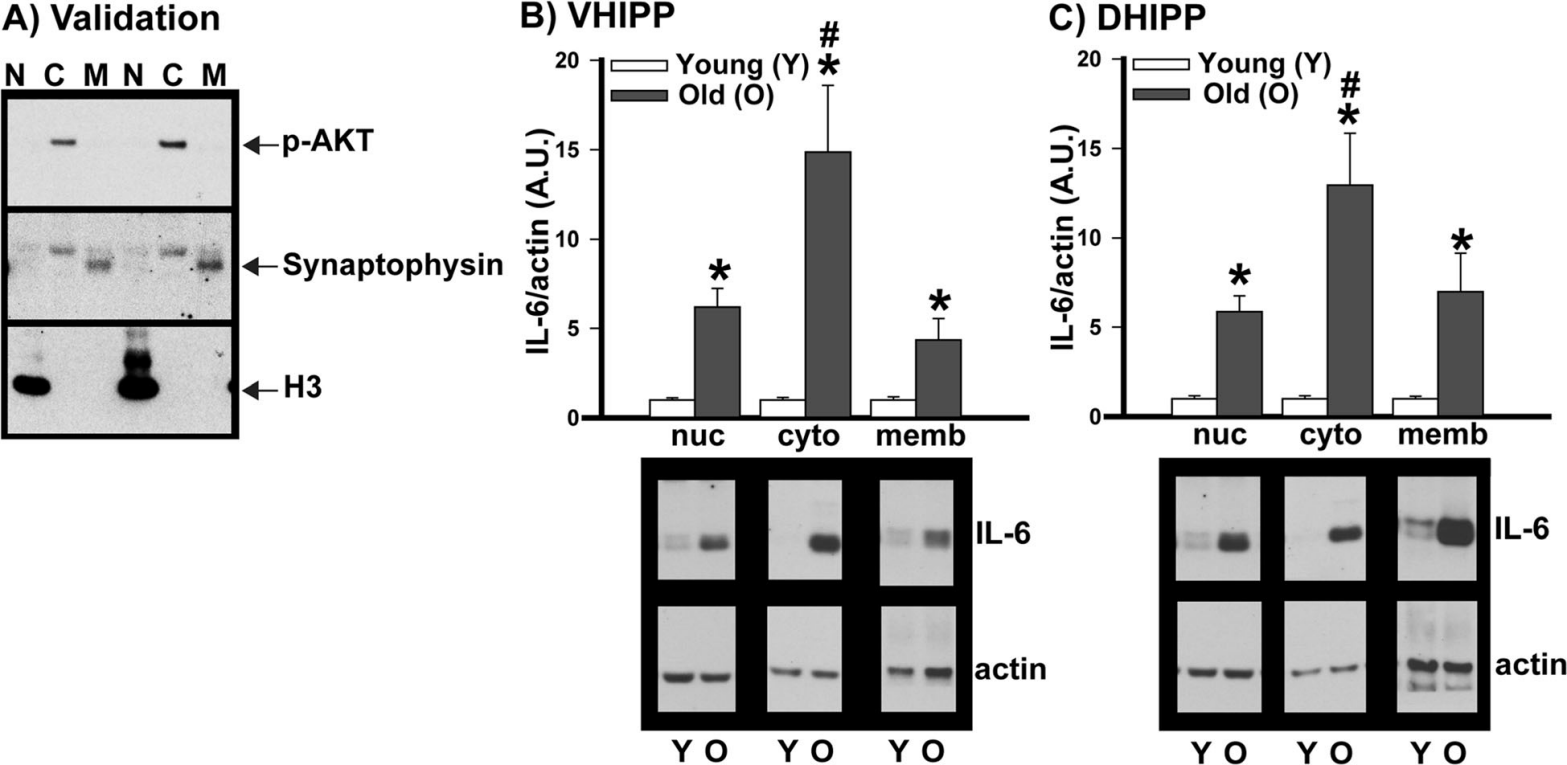
Age-related increases in hippocampal IL-6 are more pronounced in cytosolic versus nuclear or membrane fractions. To determine if age-related increases in IL-6 were more likely to impact the membrane-associated classical anti-inflammatory pathway or the soluble trans-signaling pro-inflammatory pathway, hippocampi from C57BL/6J mice were subjected to biochemical fractionation to separate proteins into nuclear (N, nuc), cytosolic (C, cyto), and membrane (M, memb) fractions. **A** Validation of biochemically fractionated samples suggests minimal contamination of the nuclear fraction with unsheared cells as we find histone 3 in the nuclear fraction but not cytosol or membrane, pAKT enriched in the cytosol fraction, and synaptophysin in the membrane but not nuclear or cytosolic fraction. **B** In the ventral hippocampus (VHIPP), old mice showed higher expression of IL-6 relative to young mice in the nuclear, cytosolic, and membrane fractions. **C** The dorsal hippocampus (DHIPP) showed the same pattern with old mice expressing higher levels of IL-6 relative to young mice in the nuclear, cytosolic, and membrane fractions. Across the ventral and dorsal hippocampus, the age-related increases that were observed in the cytosolic fractions were approximately twice those observed in the nuclear and membrane fractions. Data expressed as mean ±SEM. Brightness and contrast of blots adjusted for graphical clarity. Post hoc: #vs nuclear and membrane, *P*<0.001; *vs young, *P*<0.002

To determine if the observed age-related increases in IL-6 are conserved across mouse strains and rats, we measured IL-6 expression in the brains of young versus old BALB/cJ mice and Brown Norway Rats. Relative to young BALB/cJ mice, old BALB/cJ mice express significantly higher levels of IL-6 protein in the ventral hippocampus, dorsal hippocampus, prefrontal cortex, striatum, and cerebellum (Fig. 4A–E). Visual inspection of the data suggests the age-related increases in IL-6 may be more prominent in the female vs male BALB/cJ mice in the ventral hippocampus, PFC, and striatum, but the study was not sufficiently powered for a formal analysis of sex as a factor. Old Brown Norway Rats also showed higher expression of IL-6 protein in the PFC and cerebellum relative to young rats (Fig. 5F–G), with females showing higher IL-6 expression than males in the cerebellum (Table 1). Together, these data suggest that widespread age-related increases in IL-6 expression are conserved across species, are generally more pronounced in females, and are not tied to a single rearing environment.

### Age-related increases in IL-6 are more pronounced in cytosolic versus membrane or nuclear fractions

IL-6 can signal through multiple pathways, each of which has different functional consequences. Specifically, IL-6 signals via membrane-bound IL-6 receptors in the “classic” pathway to elicit anti-inflammatory responses and soluble IL-6 receptors in the “trans-signaling” pathway to elicit pro-inflammatory responses [51–53]. As such, we conducted biochemical fractionation on the ventral and dorsal hippocampus of young versus old C57BL/6J mice. In both ventral and dorsal hippocampus, we found age-related IL-6 increases occur in all fractions but that the magnitude of the age-related increase in cytosolic IL-6 was twice that observed in the membrane or nuclear fractions (Fig. 5; effect of a fraction within old: *F*(2,14)=13.35, *P*<0.001; post hoc: cytosolic vs. nuclear and membrane, *P*<0.001). Parsimoniously, this points to a predominantly pro-inflammatory consequence of these age-related increases.

## Discussion

Here we showed that age-related increases in cytokine expression are pervasive in the ventral hippocampus of C57BL/6 mice, with 20 out of the 23 cytokines showing significantly greater expression in aged vs. young adult mice. The 20 cytokines upregulated with age included the pro-inflammatory cytokines IL-1α, IL-1β, IL-2, IL-3, IL-6 (see further discussion below in paragraph 4), IL-9, IL-12p40, IL-12p70, IL-17, eotaxin, interfeuron δ, KC, MIP-1a, MIP-1b, rantes, and TNFα as well as the anti-inflammatory cytokines IL-4, IL-10, IL-13, and G-CSF (Fig. 1). Cytokine expression levels also became much more strongly correlated in old mice (80 significant correlations) vs young mice (9 significant correlations (Fig. 1X, Tables S2 and S3), with a unique module/network of 11 highly intercorrelated cytokines emerging in old mice (IL-1α, IL-1β, IL-3, IL-6, KL12p40, IL-13, IL17, KC, MCP-1, MIP-1b, and Rantes). It has been suggested that the identification of such disease-associated modules may be useful biomarkers for diagnosis or predicting patient outcomes and/or treatment responses (e.g., [50, 54, 55]). Further, IL-10 and IL-1β exhibited a shift from glycosylated to unglycosylated isoforms in the ventral hippocampus, suggesting even higher specific activity [56]. Interestingly, age-related increases in cytokine expression outside of the hippocampus appear to be more discreet, with the prefrontal cortex, striatum, and cerebellum demonstrating an age-related increase in IL-6 expression but not IL-1β. Importantly, we found this widespread age-related increase in IL-6 is conserved across species, occurring in C57BL/6J mice, BALB/cJ mice, and Brown Norway rats. In select brain regions, the age-related increases in IL-6 were more pronounced in females relative to males, and biochemical fractionation suggests the age-related increases in IL-6 disproportionately target pro- vs. anti-inflammatory signaling cascades. Together, our data are consistent with pathway analyses that suggest age-related changes in inflammation- and immune-related gene expression are more pronounced in the hippocampus compared to other brain regions and more pronounced in females versus males [7, 8, 57].

There have been many inconsistencies in the literature with regard to reports of age-related changes—or lack thereof—in cytokine protein expression. Our results are consistent with several past studies showing age-related increases in basal IL-1β protein in the hippocampus of a variety of naturally aging mice and rats [23–28], but not in brain regions outside of the hippocampus [22, 23, 30–32, 37]. That said, our results differ from several studies specifically using the F344xBN rats from the NIA colony that found no effect of age on basal IL-1β protein expression; although they did report age-related exacerbation of IL-1β protein induction caused by a high-fat diet, *Escherichia coli* infection or surgery [22, 30–32]. As noted above, findings around IL-6 are particularly diverse, with many studies reporting age-related increases in basal IL-6 expression in the brains of naturally aging rodents [21, 33, 40–42] and several others finding no age-related changes [22, 23, 37]. Inconsistencies in the literature around IL-6 cannot be so easily explained by differences in the strains used or the specific brain region examined. For example, one study employing C57BL/6J mice found age-related increases in IL-6 protein expression in the cortex as did we [34]; however, the effect in another study failed to reach statistical significance [23]. Such inconsistencies in the literature may be related to differential sensitivities of antibodies. Indeed, we found Femto chemiluminescence substrate was needed to reliably detect cytokine expression in our whole-tissue homogenates. Differing environmental factors across labs may also contribute to reported inconsistencies. For example, diet, and other environmental factors have been shown to preferentially upregulate cytokine expression in aged vs. young adult brains [11, 12, 35–37]. Differences in ambient temperatures from facility to facility may even be to blame since higher temperatures can increase the expression of cytokines [58] or increase the transport of cytokines from the periphery into the brain [59]. The age-related increases in IL-6 we have measured are the most robust biochemical finding ever observed in our lab. They have been detected using 2 different IL-6 antibodies, using tissue from multiple brain regions from multiple cohorts of mouse strains raised at different times in our facility and even tissue from rats raised in a different animal facility. That said, we cannot rule out the possibility that our ability to detect such reliable age-related increases in IL-6 expression may be directly related to environmental factors present in the animal facility at the University of South Carolina at the time of tissue harvesting, since the rats obtained from the NIA colony did have to habituate for 1 week prior to experimentation.

It will be of interest to future studies to determine the cell type and mechanism driving the region-specific changes in cytokine expression described herein. Microglia release a myriad of cytokines [60] but cytokines can also be released by brain endothelial cells, astrocytes, and neurons as well [42, 60, 61]. Neurons can further regulate cytokine levels by releasing signals that either activate (i.e., “on signals”) or deactivate/inhibit microglia (i.e., “off signals”) [62]. Our findings suggest that age-related increases in the hippocampus may be more extensive than those in the prefrontal cortex, striatum, or cerebellum. This increased sensitivity of the hippocampus is consistent with the fact that the hippocampus is one of the brain regions most populated with microglia [13].

Upon biochemical fractionation of the hippocampus, we found that IL-6 expression was increased with age in all fractions but that the magnitude of the increase was far greater in the cytosolic versus nuclear or membrane fraction. We cannot completely exclude the possibility that expression observed in the nuclear fraction reflects contamination by unsheared cells; however, the fact that we do not find substantial expression of the cytosolic marker pAKT or the membrane marker synaptophysin in the nuclear fractions suggests such contamination of our nuclear fraction is minimal. Even if the IL-6 expression found in the nuclear fraction actually reflects contamination from the membrane of unsheared cells, it would not change the conclusion drawn from the experiment—that is, that the magnitude of age-related increases is larger in the soluble fraction. This pattern has important functional implications. IL-6 can signal via membrane-bound IL-6 receptors in the “classic” pathway to elicit anti-inflammatory responses, or IL-6 can signal via soluble IL-6 receptors in the “trans-signaling” pathway to elicit pro-inflammatory responses [51, 52]. The fact that age-related increases in hippocampal IL-6 are far greater in the cytosolic versus membrane fraction points to a proinflammatory response. Indeed, increased activation of IL-6 trans-signaling in the brain has been implicated in several inflammatory age-related diseases of the nervous system, including Alzheimer’s and Parkinson’s Disease (c.f., [52]). The fact that we observe age-related increases in the nuclear fraction suggests IL-6 may also participate in a non-canonical cytokine signaling pathway whereby cytokine-bound receptors are internalized to the cytosol for transport to the nucleus (e.g., [63]). It will be of interest to future studies to understand if targeting soluble vs membrane IL-6 receptors may prove therapeutic for the aging brain [64, 65].

Age-related increases in pro-inflammatory cytokines are thought to be detrimental since higher expression of pro-inflammatory cytokines in humans and rodents correlates with deficits in cognitive function, synaptic plasticity, neurogenesis, and neurotrophic factor expression [9, 14, 25, 27, 30, 32, 66–73]. In contrast, an age-related increase in anti-inflammatory cytokines is thought to be protective, while their loss impairs plasticity and cognition [26, 74–77]. Here, we found that pro- and anti-inflammatory cytokines were upregulated largely in parallel with each other (Table 2). Further, the module of highly intercorrelated cytokines that emerged with age (i.e., IL-1α, IL-1β, IL-3, IL-6, KL12p40, IL-13, IL17, KC, MCP-1, MIP-1b, and Rantes) included both pro- and anti-inflammatory pathway members. Together, this suggests an attempt of anti-inflammatory pathways to compensate for the dysregulation of pro-inflammatory pathways. It is likely that the widespread age-related increase in IL-6 noted herein is detrimental since the majority of studies suggest elevated expression impairs cognitive function ([66, 67, 78, 79], but see [42]). As such, therapeutics that restore cytokine signaling may prove beneficial in the treatment of age-related disorders.

It is noteworthy that robust and consistent sex differences were observed in the present study [80]. Whereas the female-aged brain was biased towards exaggerated inflammation relative to the male brain, no differences between males and females were evident in young or middle-aged mice. Although the majority of these studies were not powered to allow for statistical sex-based conclusions, the exacerbated neuroinflammation observed within the aging female brain was reliable and robust. For example, 14/17 old females exhibited VHIPP IL-6 levels that exceeded the mean value measured in old males. These data are in line with other studies indicating that neuroinflammation accumulates in the aged female hippocampus to a greater extent than males [57] and highlights a potential mechanism whereby females display faster age-related cognitive decline than men [81] and higher rates of Alzheimer’s disease [82]. It will be of interest to future studies to understand the basis for these augmented female responses. Both female and male hormones fluctuate over time and change with age in rodents, with age-related decreases in pulsatile GnRH observed in male rodents and a multiplicity of ovarian states observed in aging female rodents (i.e., estropause in a persistent estrus phase, estropause in a persistent diestrus phase, or irregular cycling) [83–87]. Such changes in ovarian aging status have been shown to influence inflammatory and metabolic gene expression in the rat hippocampus, albeit not differentially in the ventral versus dorsal hippocampus [88]. Further, a number of peripheral inflammatory diseases increase with time—some of which occur differentially in males versus females and some of which elicit different cytokine profiles in males versus females (e.g., [89, 90]). The fact that we did not track hormonal status or peripheral pathology may be considered a weakness of the present study. That said, it is not intuitive how global changes in blood cytokine levels triggered by hormones or tumors would elicit such brain region-specific effects (i.e., ventral but not dorsal hippocampus) or cytokine-specific sex effects (i.e., IL-6 but not IL-1 or IL-10). Whatever their basis, efforts to therapeutically target cytokine signaling should give serious consideration to these sex differences, particularly given that anti-inflammatory therapeutic responses [91] and the half-life of cytokine antagonists [92] differ in males versus females.

A number of therapeutic avenues are currently being pursued to target age-related increases in neuroinflammation. Natural products containing resveratrol have been shown to attenuate serum IL-6 and TNFα levels in healthy older adults along with memory retention and hippocampal functional connectivity [93, 94]. Other antioxidants have reduced TNFα and IL-1β mRNA and protein expression in the brains of senescence-accelerated mouse models [95]. Reported effects of melatonin are mixed with 1 study reporting reduced TNFα, IL-1β, and IL-6 protein expression in the hippocampus [21] and the other reporting no effect on age-related increases in TNFα and worsening of age-related increases in brain IL-1α [96]. Probiotics not only lowered TNFα and MCP1 protein expression in the serum while increasing IL-10 protein expression, but also improved cognition in a senescence-accelerated mouse model (males and females, [97]). Biological approaches have also been taken to overcome the damaging effects of pro-inflammatory cytokines, including infusion of receptor antagonists or anti-inflammatory cytokines [26, 28, 32, 69, 74, 76, 77, 98]. Of particular note—given our biochemical fractionation data—biologics that inhibit IL-6 trans-signaling specifically are being developed in the context of a number of inflammatory diseases (c.f., [52]). Finally, behavioral therapeutic approaches, such as mindfulness training or exercise, have also shown promise in attenuating age-related increases in IL-1β and IL-6, at least in males [99–102] and cognitive behavioral therapy has been shown to boost immune function across sexes by reducing proinflammatory molecules and improving immune cell counts [103]. Importantly, peripheral markers of inflammation and immune activation may prove viable patient-selection biomarkers for such clinical trials given that several studies demonstrate parallel changes in the brain and blood or saliva [7, 8, 33] as well as correlations between elevated cytokine expression in the serum and reduced cognitive function [104].

## Conclusion

Taken together, our findings suggest that age-related increases in cytokines are more pronounced in the hippocampus compared to other brain regions and can be more pronounced in females versus males depending on the brain region, genetic background, and cytokine examined. As such, it will be important to consider sex differences in the underlying pathology as well as pharmacokinetics/pharmacodynamics when considering cytokines as therapeutic targets in the context of age-related disease.

## Supplementary Information

**Additional file 1: Figure S1.** Left: Indication of how western blot membranes are cut after ponceau stain but prior to blocking and incubating with primary antibody. Right: Overexposed film shown to enable visualization of the edges of the cut membranes (cut edges indicated by scissor icons). **Figure S2.** Unadjusted images of blots presented in Figure 2A. Age inversely correlates with ID number (i.e., youngest mice are those born later and thus have the highest ID). **Figure S3.** Unadjusted images of blot presented in Figure 2B-C. Y—young, M—middle age, O—old, X—+/- control samples for other antibodies used to probe membrane at a higher molecular weight (not a part of this study). **Figure S4.**Unadjusted images of blots presented in Figure 3A-D. Y—young, M—middle age, O—old, X—+/- control samples for other antibodies used to probe membrane at a higher molecular weight (not a part of this study). **Figure S5.** Unadjusted images of blots presented in Figure 3E-H. Y—young, M—middle age, O—old, X—+/- control samples for other antibodies used to probe membrane at a higher molecular weight (not a part of this study). **Figure S6.** Unadjusted images of blots presented in Figure 4A-E. Y—young, O—old. **Figure S7.** Unadjusted images of blots presented in Figure 4F-G. Y—young, O—old. **Figure S8.** Unadjusted images of blots presented in Figure 5. Y—young, O—old, X—+/- control samples for other antibodies used to probe membrane at a higher molecular weight (not a part of this study). **Figure S9.** Data from Figure 1 replotted by sex. The bar placed above the old males and females with the asterisk on top is intended to reflect the main effect of age (i.e., there were insufficient n/sex to warrant an analyses of age x sex). **Figure S10.** Ratios of glycosylated/unglycosylated cytokines exhibit age-related changes in select brain regions. IL-10 and IL-1β data from Figures 2 and 3 were re-expressed as a ratio of the density of the top band (i.e., presumed glycosylated isoform) over the density of the bottom band (i.e., presumed unglycosylated isoform). A) In ventral hippocampus (VHIPP), the ratio of glycosylated/unglycosylated IL-10 decreased with age (effect of age: F(2,28)=21.19, P<0.001; Post hoc: young vs. middle P=0.002, young vs. old P<0.001, and middle vs. old P=0.033 ). Although this shift was somewhat more pronounced in females, the effect did not reach the level of statistical significance (effect of sex: F(1,28)=3.30, P=0.08). B) In VHIPP, the ratio of glycosylated/unglycosylated IL-1β similarly decreased with age (effect of age: F(1,23)=16.09, P<0.001). In contrast, the ratio of glycosylated/unglycosylated IL-1β remained stable in C) dorsal hippocampus (DHIPP) and D) prefrontal cortex (PFC), and E) actually increased in striatum of females (2-Way ANOVA failed normality; Rank Sum Test females: T(6,7)=27.00, P=0.035; student t-test males: t(20)=0.03, P=0.98). *vs. young only, P=0.002 to <0.001; #vs young and middle, P=0.033 to <0.001.

**Additional file 2: Table S1.** Cytokine expression detected in young adult, middle-aged and old ventral hippocampus (n= 8/group) when using 0.275 mg total protein in the Biorad Bioplex assay.

**Additional file 3: TableS2.** Correlational analyses of Bioplex data shown in Figure 1 reveals cytokines are largely uncorrelated in ventral hippocampus of young mice (shown right; significant FDR-P values bolded) but approximately half of these cytokines become highly correlated with each other in old mice (shown left; gray highlighting indicates correlations that are significant in both old and young mice).

**Additional file 4: Table S3.** A chart of results from correlational analyses shown in Table S2 (significant r, 1; non-significant r. 0) reveals a module of 11 highly intercorrelated cytokines (highlighted in yellow; red text indicates rare failures in module members correlating with each other).

## Data Availability

The datasets used and/or analyzed during the current study are available from the corresponding author on reasonable request.

## Abbreviations

FDR: False rate discovery
G-CSF: Granulocyte colony-stimulating factor
GM-CSF: Granulocyte-macrophage colony-stimulating factor
IL: Interleukin
IFN: Interfeuron
MCP1: Monocyte chemoattractant protein 1
MIP: Macrophage inflammatory protein
PFC: Prefrontal cortex
TNF: Tumor necrosis factor
