# Supplementary material for: Aging triggers an upregulation of a multitude of cytokines in the male and especially the female rodent hippocampus but more discrete changes in other brain regions
Source: J Neuroinflammation. 2021 Sep 22;18:219. doi: 10.1186/s12974-021-02252-6 (PMC8459490; doi:10.1186/s12974-021-02252-6)
Supplement: Supplementary file 1 — Additional file 1: Figure S1. Left: Indication of how western blot membranes are cut after ponceau stain but prior to blocking and incubating with primary antibody. Right: Overexposed film shown to enable visualization of the edges of the cut membranes (cut edges indicated by scissor icons). Figure S2. Unadjusted images of blots presented in Figure 2A. Age inversely correlates with ID number (i.e., youngest mice are those born later and thus have the highest ID). Figure S3. Unadjusted images of blot presented in Figure 2B-C. Y—young, M—middle age, O—old, X—+/- control samples for other antibodies used to probe membrane at a higher molecular weight (not a part of this study). Figure S4.Unadjusted images of blots presented in Figure 3A-D. Y—young, M—middle age, O—old, X—+/- control samples for other antibodies used to probe membrane at a higher molecular weight (not a part of this study). Figure S5. Unadjusted images of blots presented in Figure 3E-H. Y—young, M—middle age, O—old, X—+/- control samples for other antibodies used to probe membrane at a higher molecular weight (not a part of this study). Figure S6. Unadjusted images of blots presented in Figure 4A-E. Y—young, O—old. Figure S7. Unadjusted images of blots presented in Figure 4F-G. Y—young, O—old. Figure S8. Unadjusted images of blots presented in Figure 5. Y—young, O—old, X—+/- control samples for other antibodies used to probe membrane at a higher molecular weight (not a part of this study). Figure S9. Data from Figure 1 replotted by sex. The bar placed above the old males and females with the asterisk on top is intended to reflect the main effect of age (i.e., there were insufficient n/sex to warrant an analyses of age x sex). Figure S10. Ratios of glycosylated/unglycosylated cytokines exhibit age-related changes in select brain regions. IL-10 and IL-1β data from Figures 2 and 3 were re-expressed as a ratio of the density of the top band (i.e., presumed glycosylated isoform) over the density of the botto [file 12974_2021_2252_MOESM1_ESM.pdf]

Supplemental Figures for ***Aging triggers an upregulation of a multitude of cytokines in the male and especially the female rodent hippocampus but more discrete changes in other brain regions***

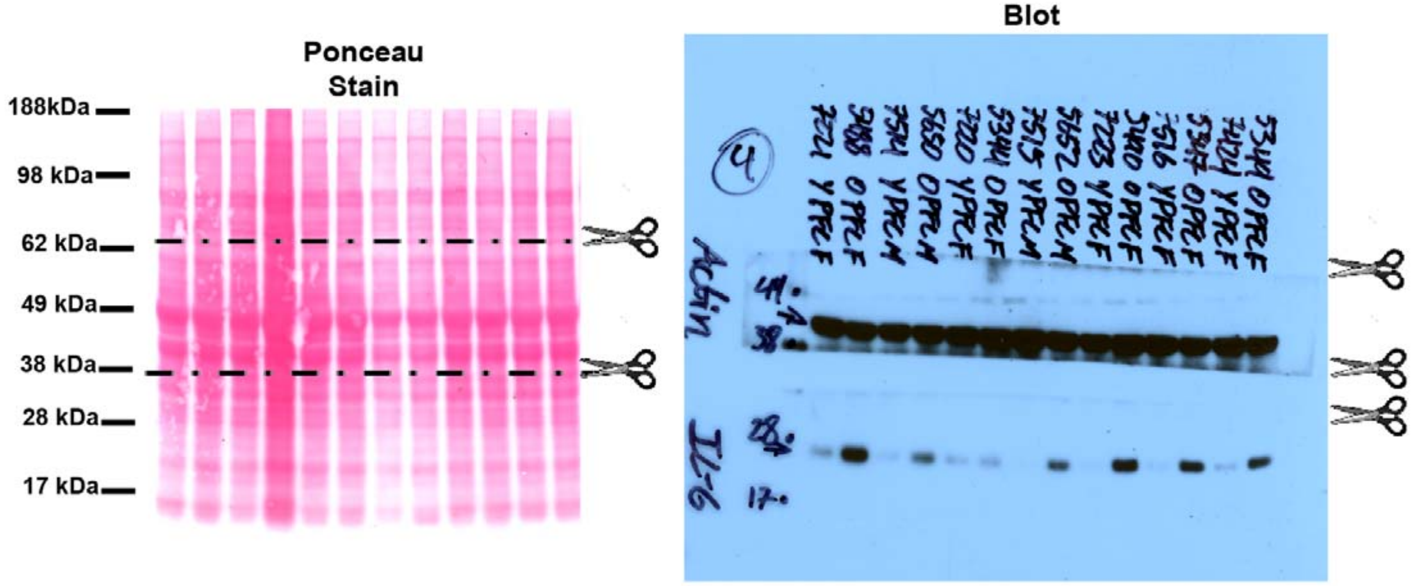

Figure S1. Left: Indication of how western blot membranes are cut after ponceau stain but prior to blocking and incubating with primary antibody. Right: Overexposed film shown to enable visualization of the edges of the cut membranes (cut edges indicated by scissor icons).

Full images from Fig 2A IL-10 males

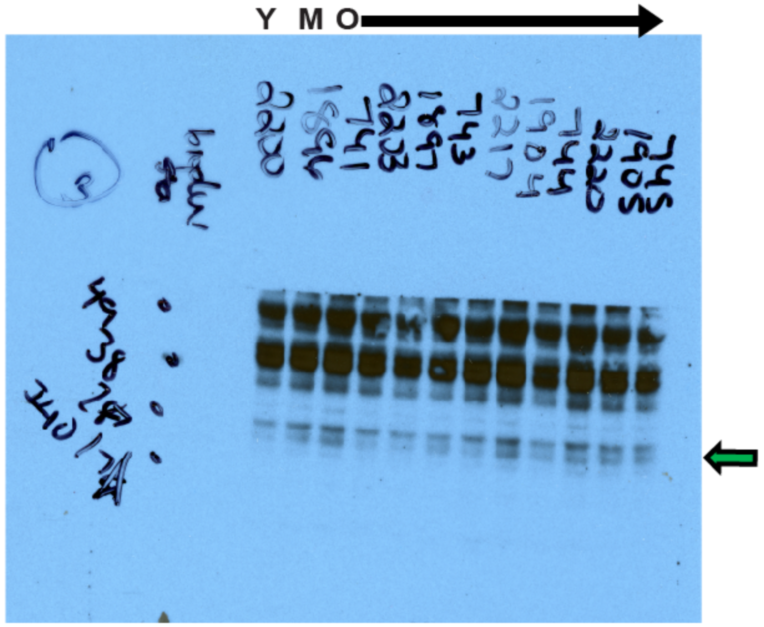

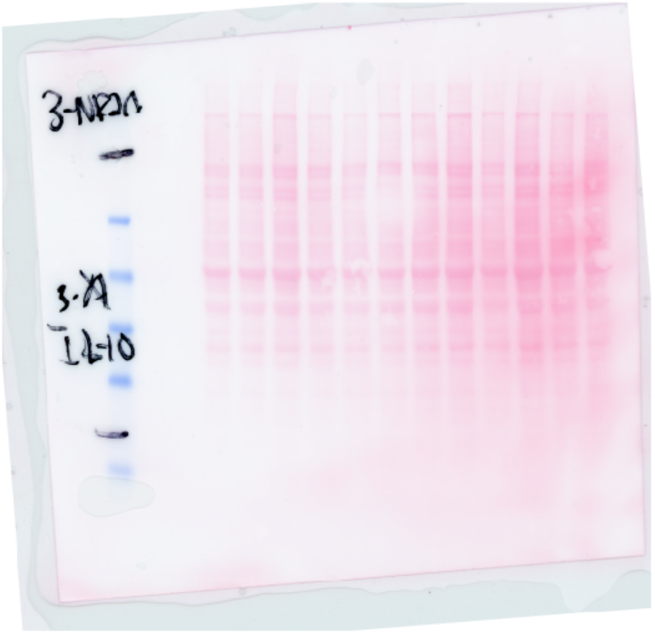

Full images from Fig 2A IL-10 females

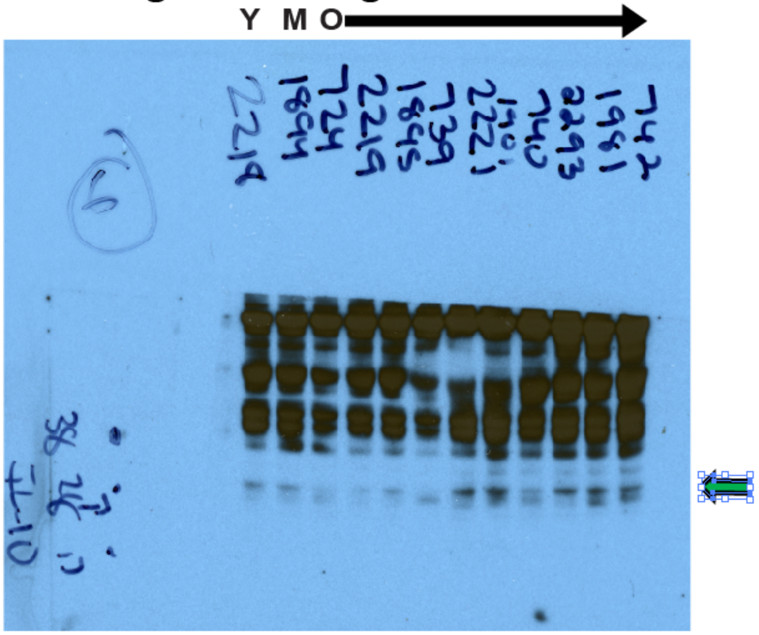

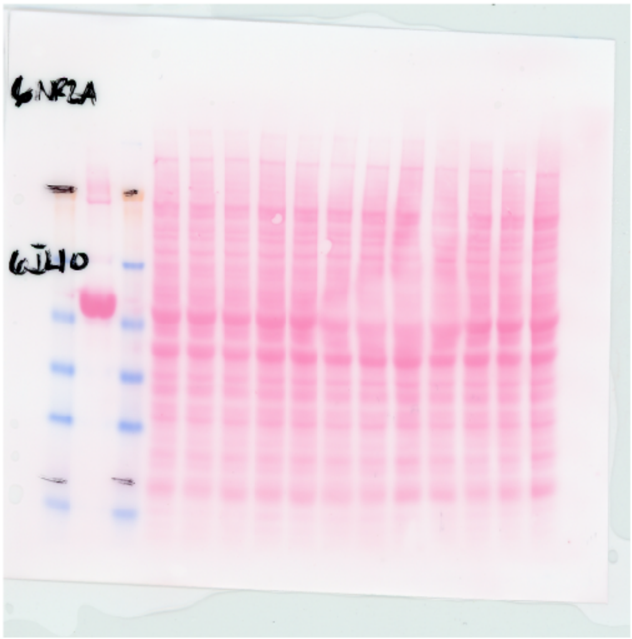

Figure S2. Unadjusted images of blots presented in Figure 2A. Age inversely correlates with ID number (i.e., youngest mice are those born later and thus have the highest ID).

Full images from Fig 2B, males & females on same blot

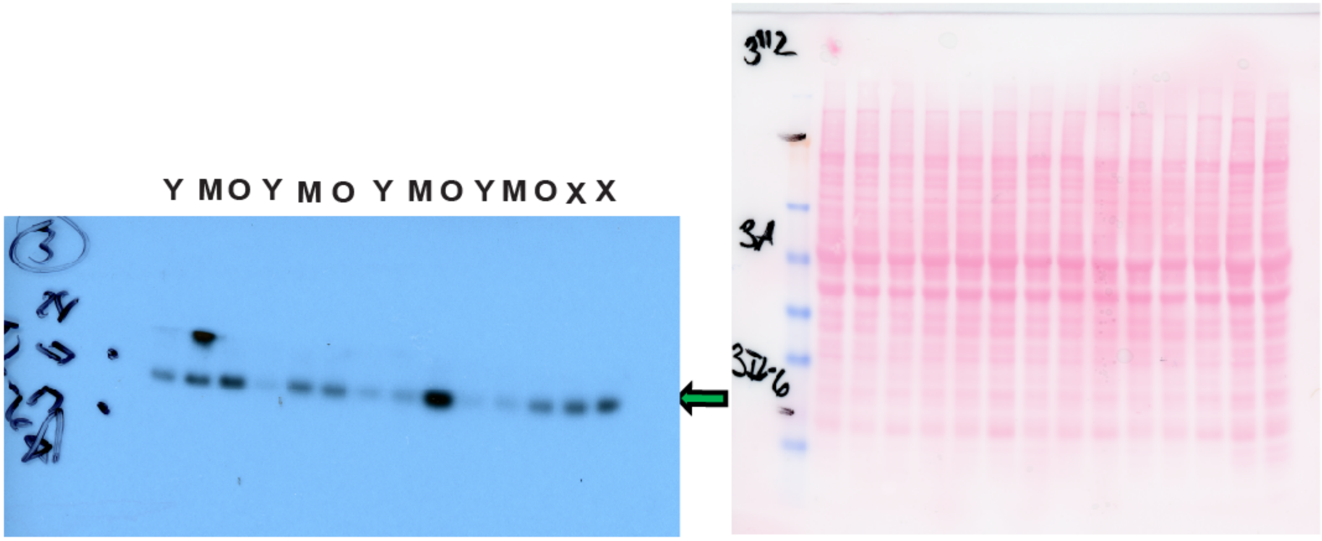

Full Images from Fig 2C males & females on same blot

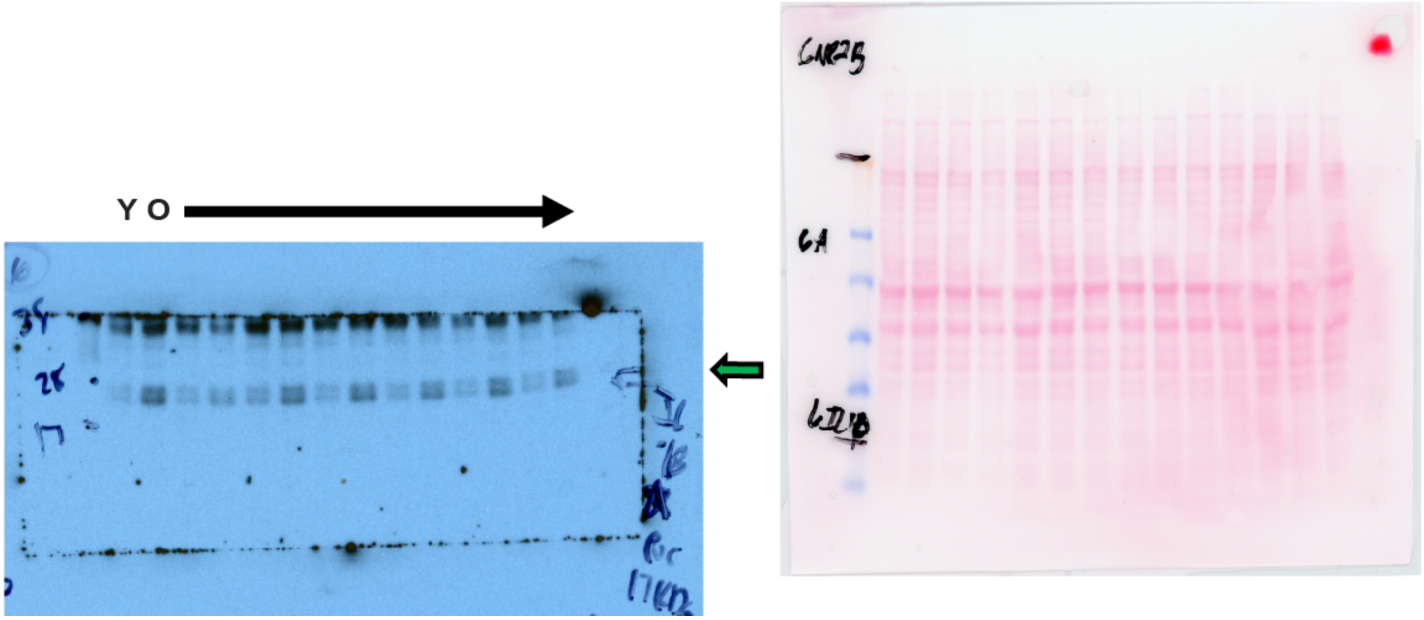

Figure S3. Unadjusted images of blot presented in Figure 2B-C. Y—young, M—middle age, O—old, X—+/- control samples for other antibodies used to probe membrane at a higher molecular weight (not a part of this study).

Full images from Fig3A DHIPP IL-6

Y M O Y M O Y M O Y M O X X

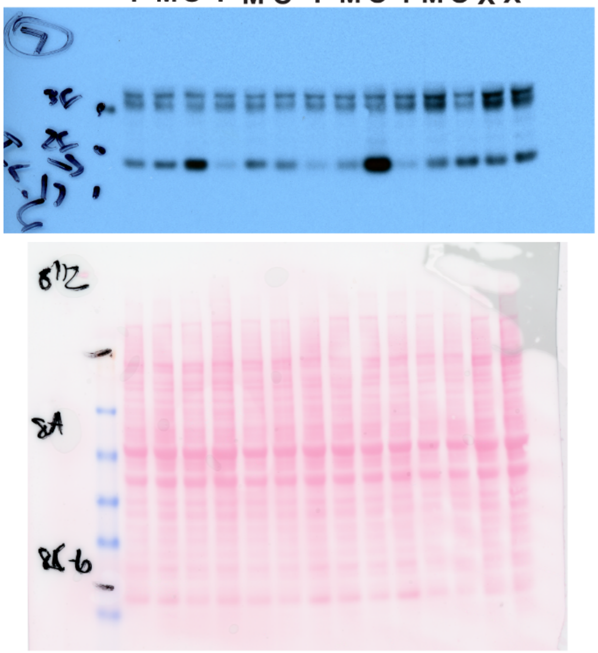

Full images from Fig3B DHIPP IL-1β

Y O Y O Y x O Y O Y O Y O Y

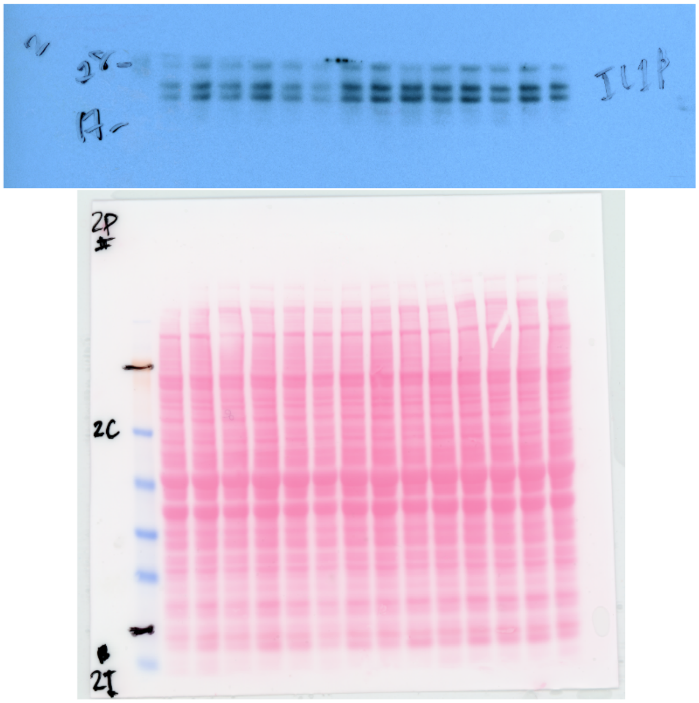

Full images from Fig3C PFC IL-6

O Y O Y O Y Y O Y O Y O Y Y

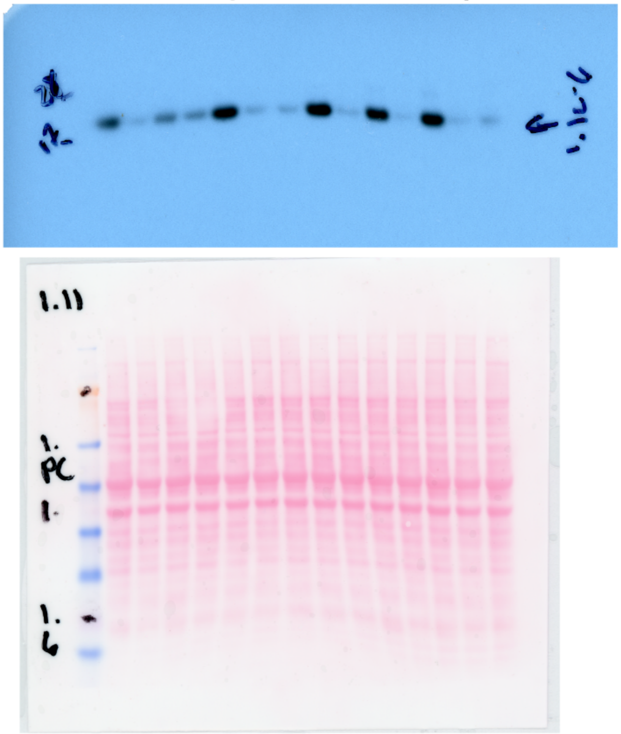

Full images from Fig3D PFC IL-1β

Y O →

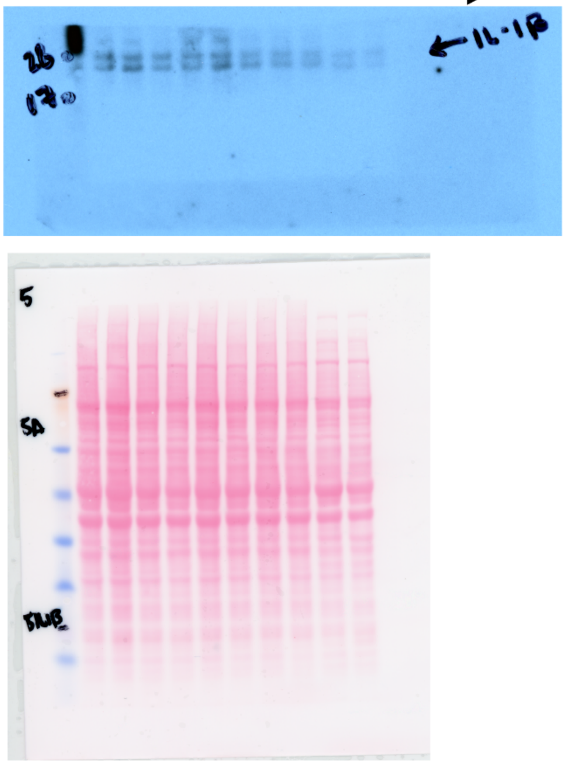

Figure S4. Unadjusted images of blots presented in Figure 3A-D. Y—young, M—middle age, O—old, X—+/- control samples for other antibodies used to probe membrane at a higher molecular weight (not a part of this study).

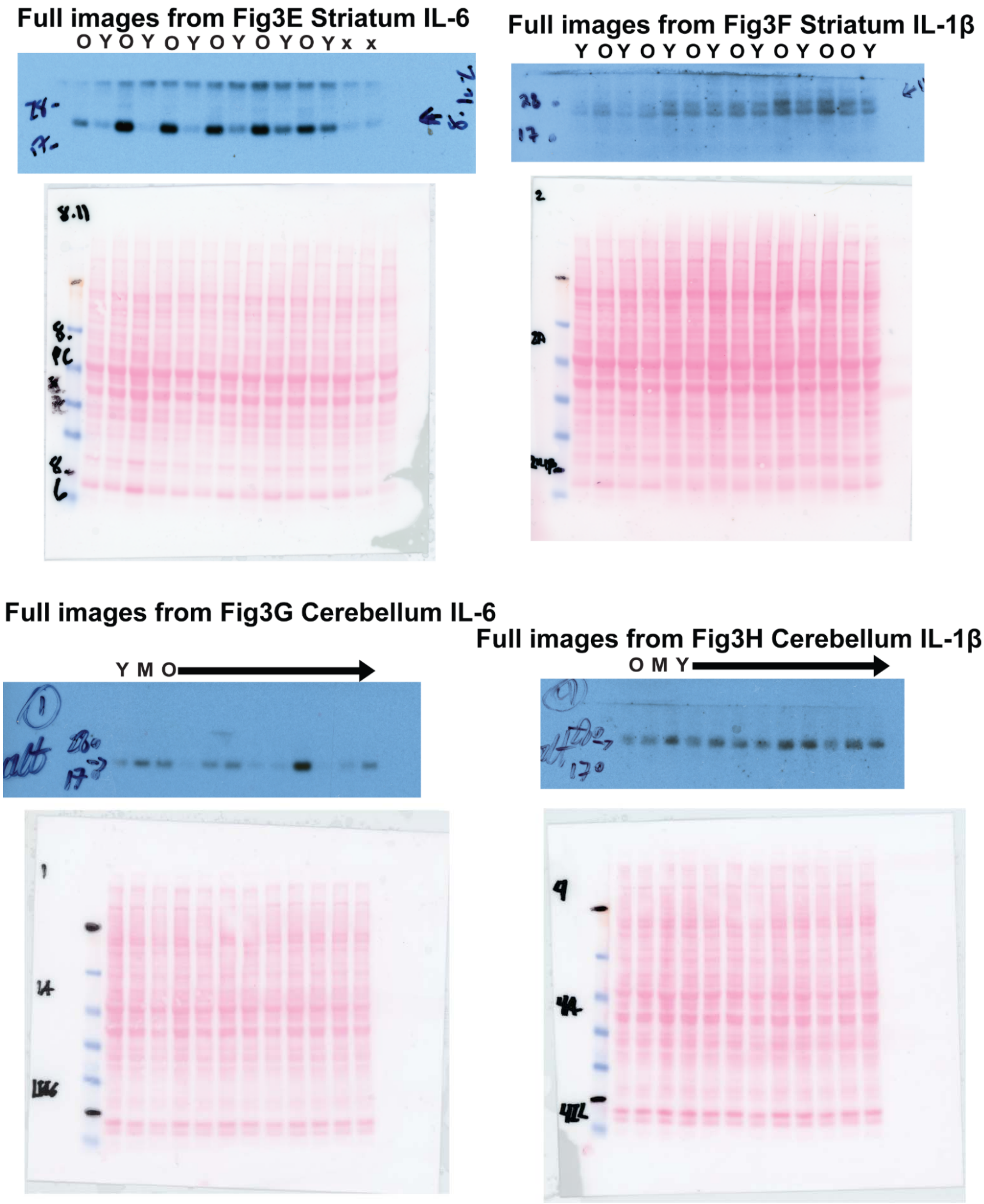

Figure S5. Unadjusted images of blots presented in Figure 3E-H. Y—young, M—middle age, O—old, X—+/- control samples for other antibodies used to probe membrane at a higher molecular weight (not a part of this study).

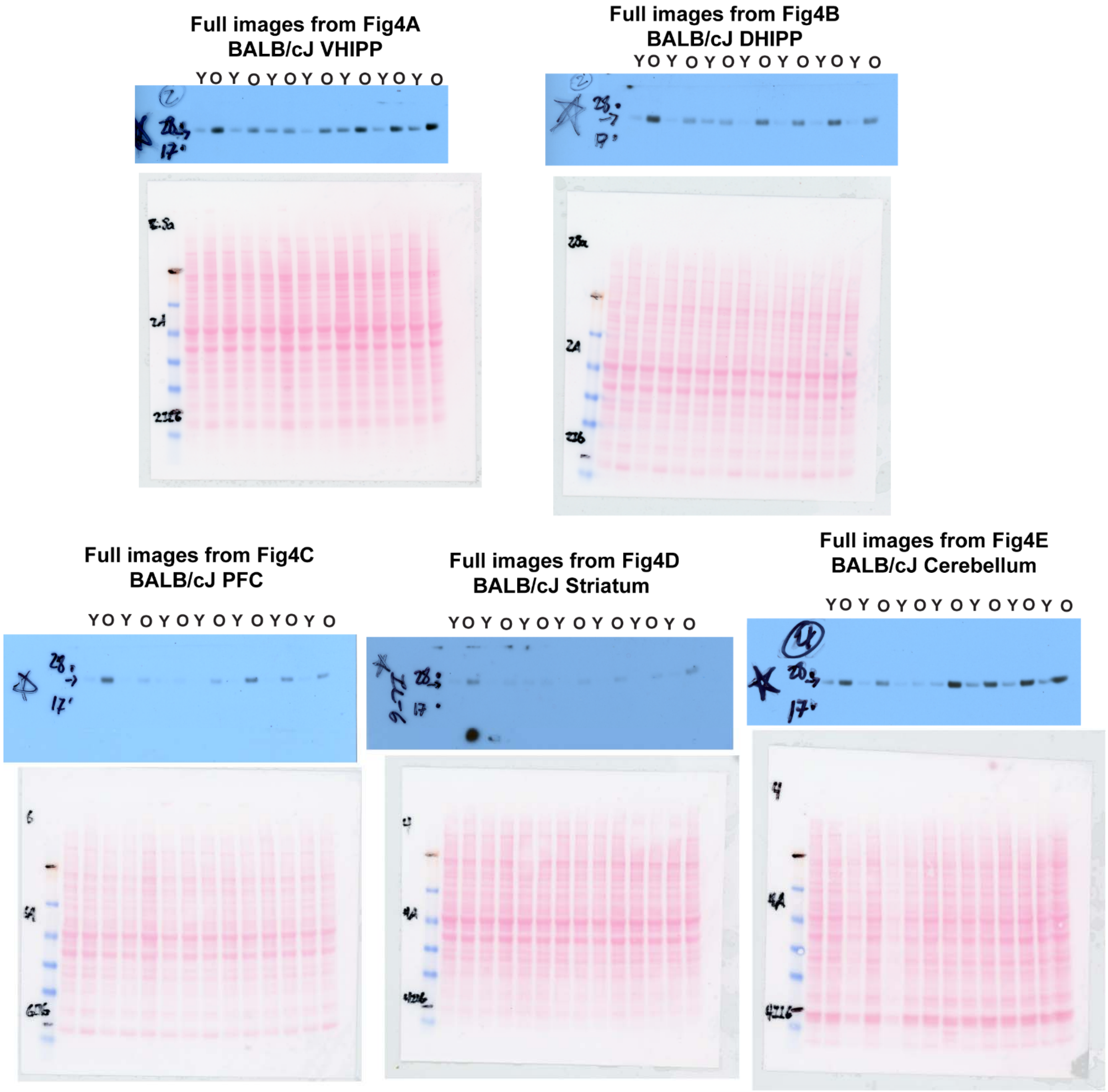

Figure S6. Unadjusted images of blots presented in Figure 4A-E. Y—young, O—old.

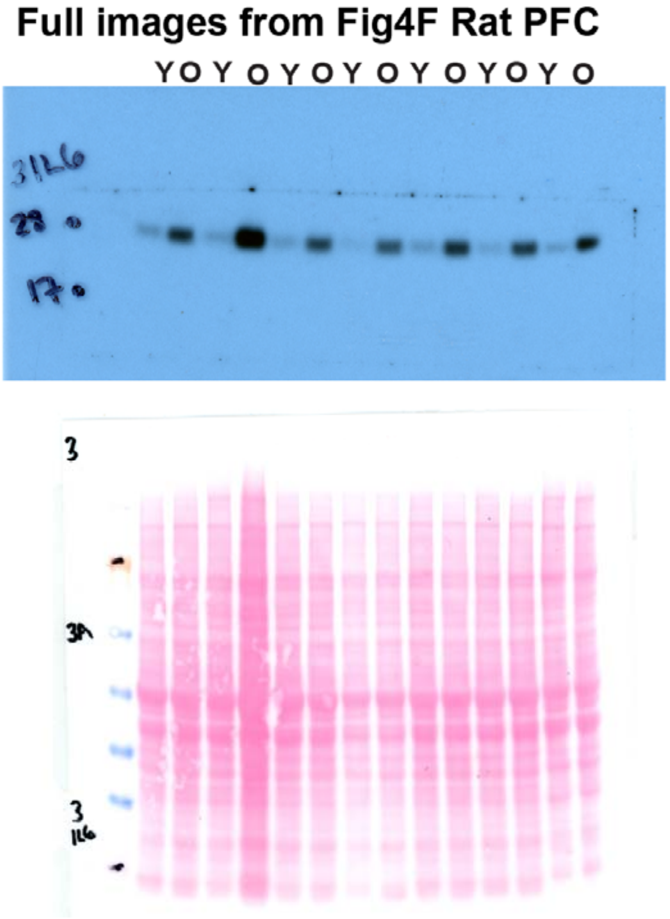

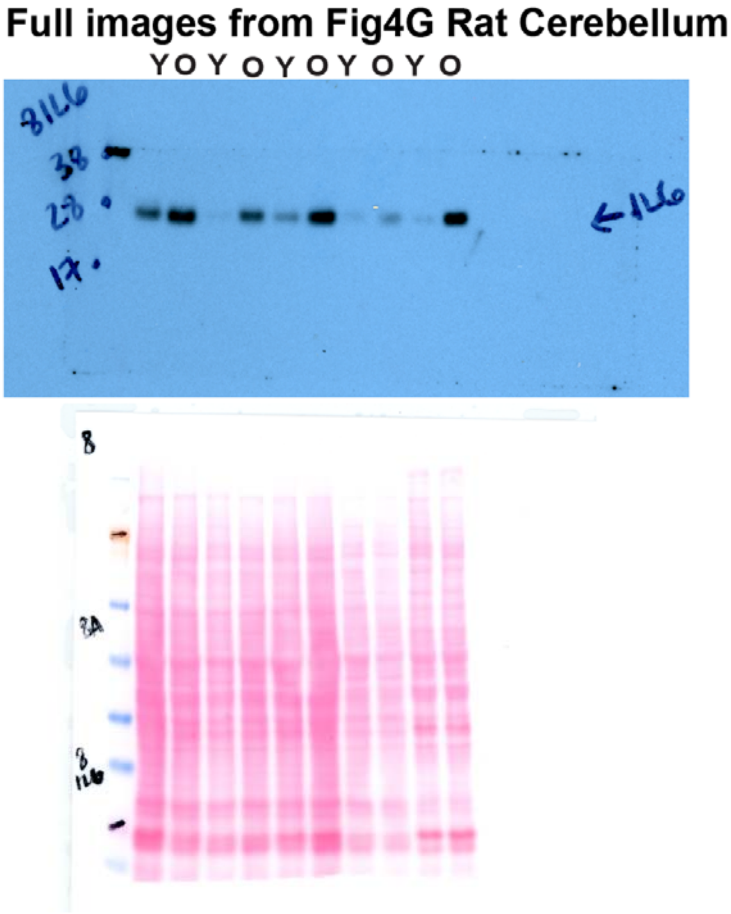

Figure S7. Unadjusted images of blots presented in Figure 4F-G. Y—young, O—old.

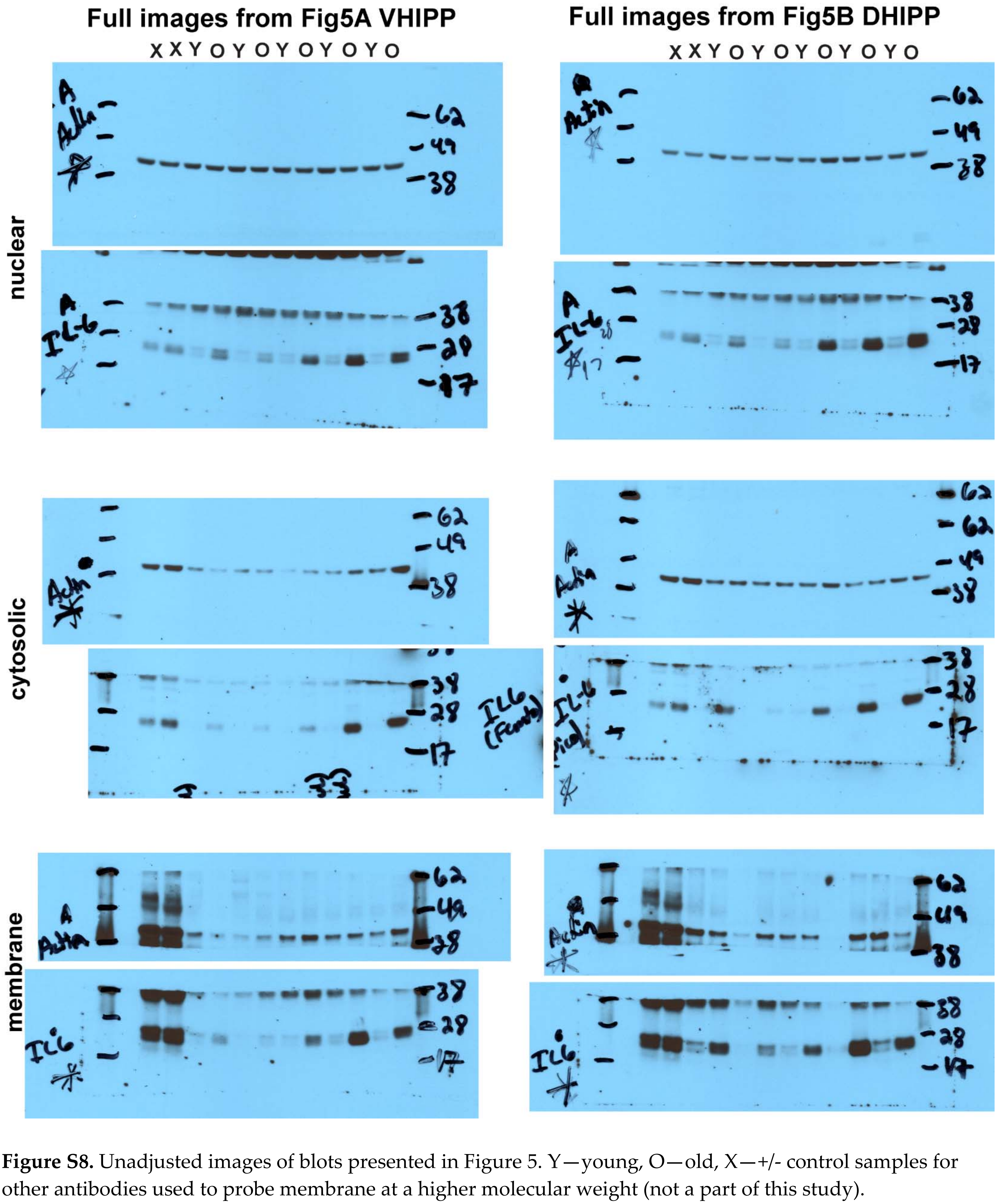

Figure S8. Unadjusted images of blots presented in Figure 5. Y—young, O—old, X—+/- control samples for other antibodies used to probe membrane at a higher molecular weight (not a part of this study).

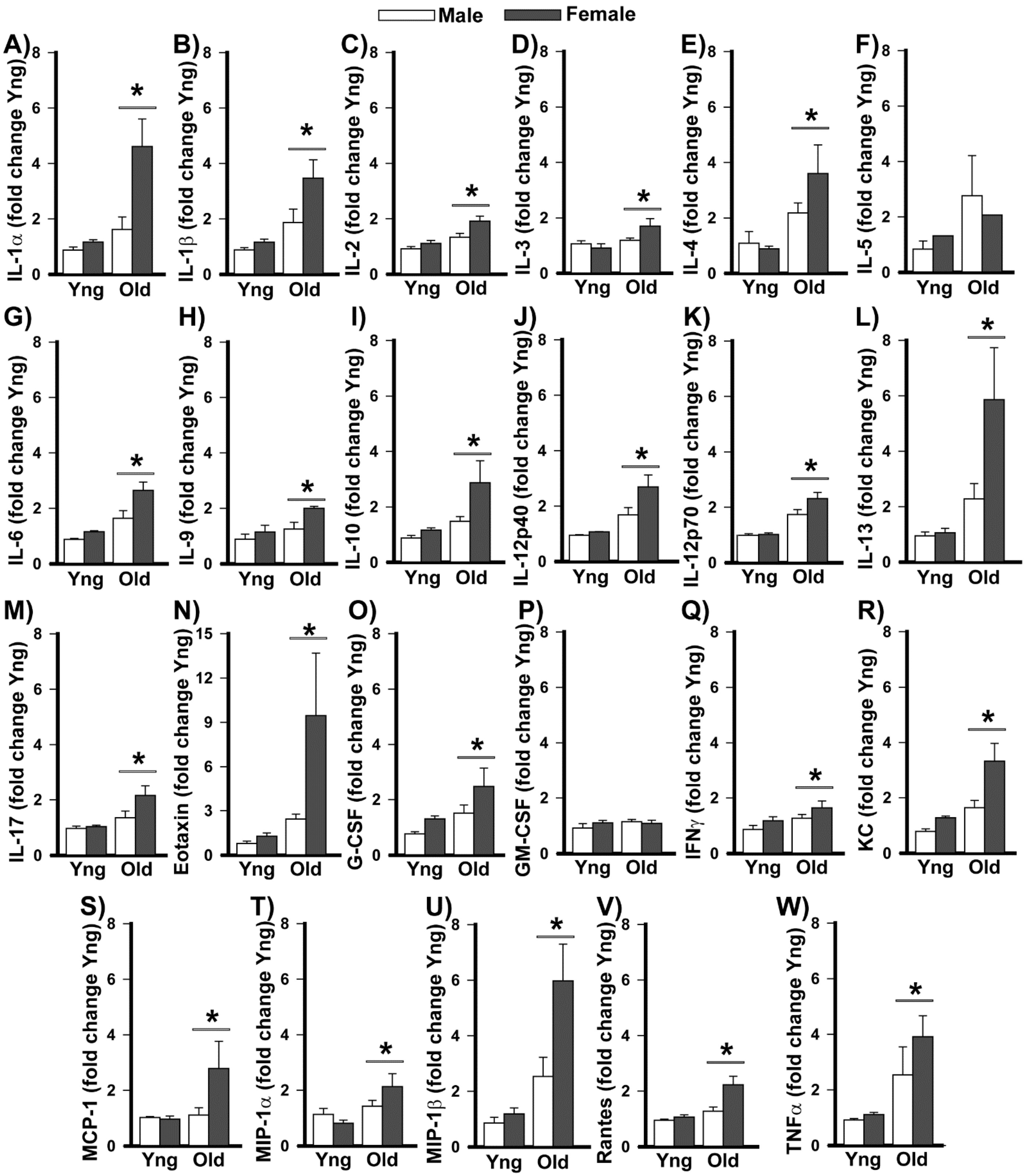

Figure S9. Data from Figure 1 replotted by sex. The bar placed above the old males and females with the asterisk on top is intended to reflect the main effect of age (i.e., there were insufficient n/sex to warrant an analyses of age x sex).

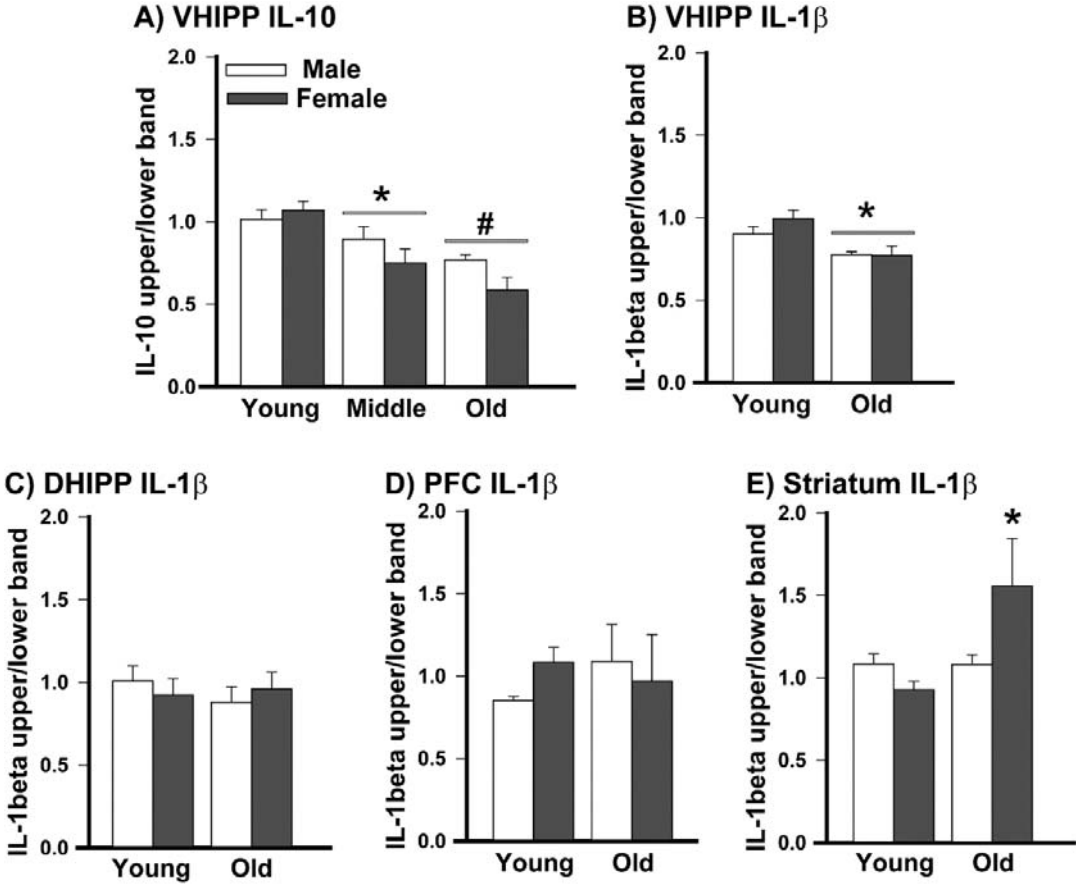

Figure S10. Ratios of glycosylated/unglycosylated cytokines exhibit age-related changes in select brain regions. IL-10 and IL-1β data from Figures 2 and 3 were re-expressed as a ratio of the density of the top band (i.e., presumed glycosylated isoform) over the density of the bottom band (i.e., presumed unglycosylated isoform). A) In ventral hippocampus (VHIPP), the ratio of glycosylated/unglycosylated IL-10 decreased with age (effect of age: $F(2,28)=21.19$, $P<0.001$; *Post hoc*: young vs. middle $P=0.002$, young vs. old $P<0.001$, and middle vs. old $P=0.033$). Although this shift was somewhat more pronounced in females, the effect did not reach the level of statistical significance (effect of sex: $F(1,28)=3.30$, $P=0.08$). B) In VHIPP, the ratio of glycosylated/unglycosylated IL-1β similarly decreased with age (effect of age: $F(1,23)=16.09$, $P<0.001$). In contrast, the ratio of glycosylated/unglycosylated IL-1β remained stable in C) dorsal hippocampus (DHIPP) and D) prefrontal cortex (PFC), and E) actually increased in striatum of females (2-Way ANOVA failed normality; Rank Sum Test females: $T(6,7)=27.00$, $P=0.035$; student t-test males: $t(20)=0.03$, $P=0.98$). *vs. young only, $P=0.002$ to <0.001; #vs young and middle, $P=0.033$ to <0.001.
